# Beyond prosociality: Recalling many types of moral behavior produces positive emotion

**DOI:** 10.1371/journal.pone.0277488

**Published:** 2022-11-11

**Authors:** Andrew Miles, Laura Upenieks, Christos Orfanidis

**Affiliations:** 1 Department of Sociology, University of Toronto, Toronto, ON, Canada; 2 Department of Sociology, Baylor University, Waco, TX, United States of America; 3 Ontario Institute for Studies in Education, University of Toronto, Toronto, ON, Canada; Sapienza University of Rome, ITALY

## Abstract

Prosocial acts can increase positive emotions and contribute to emotional well-being, but it is unclear whether other types of moral behavior have similar effects. Respondents from a large online sample (N = 1783) were randomly assigned to recall recent instances when they had performed moral, self-indulgent, or routine acts. Those who recalled self-indulgent behaviors or acts associated with care, fairness, loyalty, authority, and sanctity-based morality increased in positive emotions relative to routine acts controls. Initial evidence suggests that effects for recalling moral acts occurred in part because individuals who recalled these behaviors generated positive moral self-appraisals and satisfied a basic psychological need for relatedness. Study results are consistent with the recent claim that morality is a basic psychological need.

## Introduction

Kind people are happy people. Acts intended to benefit others—known as prosocial behaviors—have been shown to lastingly increase positive emotions in samples of various ages and nationalities, and might reduce symptoms of anxiety and depression [1–3]. The ubiquity of these effects suggests that prosocial behaviors could form the basis of low-cost, easy-to-implement interventions aimed at safeguarding emotional health. However, it is not clear whether these emotional benefits extend beyond prosociality to *any act* that a person considers moral. If so, the range of behaviors available to foster emotional well-being increases accordingly.

Scholars have generally explained prosocial effects as the natural result of fulfilling basic psychological needs for autonomy, competence, and relatedness (hereafter ACR needs) [4, 5]. However, emerging evidence suggests that there might be a fourth need that helps explain prosocial effects: *a need to feel moral* [6, 7]. Prosocial behavior might facilitate moral need satisfaction by encouraging people to see themselves as moral individuals. For instance, in a series of studies Miles and Upenieks [8] showed that recalling or performing prosocial behaviors leads to large increases in *moral self-appraisals*, which they defined as cognitive assessments of how well an individual is living up to his/her moral commitments ([9], who refer to these as “moral self-image”). These appraisals, in turn, predicted increases in positive affect even when controlling for the satisfaction of ACR needs.

The idea that morality might be essential for human flourishing has deep roots in the psychological literature. For instance, William James [10] argued that “the joy of moral self-approbation…[may be] required to make the notion of mere existence tolerable.” More recently, Steele [11] proposed in his self-affirmation theory that people are intrinsically motivated to believe that they are good. A number of studies offer evidence consistent with the claim that morality is a need. For instance, people are less likely to engage in moral behavior when their own morality is highlighted, a fact which might imply that their moral need has already been satiated [12]. Recent work by Goodwin and colleagues suggests that moral character powerfully shapes the impressions people form about others [13, 14]. This, in turn, implies that individuals are motivated to be judged favorably on moral standards, and might explain why people indicate that morality is more important to their sense of self and identity than their personality traits or autobiographical memories [15].

While some theory and research in psychology is consistent with the idea that morality is a basic psychological need, establishing this claim directly requires an extensive body of evidence [16, 17]. Here, we mention just one of the many criteria that have been offered for establishing a need, largely because it has informed early-stage tests of the morality-as-a-need hypothesis. This criterion holds that satisfying a basic psychological need should promote emotional well-being universally—that is, regardless of a person’s developmental stage or subjective preferences—while its frustration should generate emotional dysfunction. A few studies have started to examine the potential of morality to meet this requirement. Prentice and colleagues [6] conducted an empirical “entrance exam” on morality by asking both online respondents and students to recall events in which they felt satisfied or unsatisfied, at their best or worst, and that they experienced as pleasurable and meaningful. They then rated the extent to which candidate psychological needs were satisfied during those events. They found that morality was as frequently or more satisfied than needs for autonomy, competence, or relatedness during events related to satisfaction, meaning, pleasure, or being “at one’s best.” Conversely, morality was not well-satisfied during events where individuals described being at their worst. Morality was also a unique predictor of positive functioning, including flourishing and satisfaction with life, above and beyond the ACR needs. Prentice and colleagues further documented that where the satisfaction of the moral need was low, respondents reported lower positive affect and quality of life. In a follow-up study, Prentice and colleagues [7] used experience sampling to examine the effects of satisfying the moral need in daily life. They found that moral behaviors predicted positive moral self-appraisals, which in turn predicted psychological thriving as much or more than the satisfaction of ACR needs. It is noteworthy that people who reported the lowest levels of moral character at the outset of the study benefitted the most from enacting moral behavior, perhaps reflecting a desire for such experiences.

Past research thus provides indications that morality is a need, but the evidence remains limited. There are several additional steps that can be taken to help determine whether morality is a need, though as noted above fully validating this claim will require much more evidence than we are able to provide here [16, 17]. First, in a recent review, Jayawickreme, Prentice, and Fleeson [18] called for research on “which specific moral virtues best predict moral need satisfaction” (pg.112). This suggests an important implication of the morality-as-a-need perspective: that positive emotions should occur whenever a person performs *any* behavior that s/he regards as moral [7, 8]. However, work to date has focused almost exclusively on the emotional consequences of prosocial action. Prosocial action captures one important aspect of morality—caring—but a large body of evidence demonstrates that people worldwide also attach moral significance to issues such as fairness and honesty, loyalty to ingroup members, respect for legitimate authority, and (metaphorical) sanctity [19–21].

This raises the question: Does living up to any moral principle confer the same emotional benefits as acting in a prosocial way? If so, the case for morality as a need becomes stronger. Research to date suggests that the answer to this question might be yes. For example, honest individuals report less depression and greater happiness, life-satisfaction, sense of purpose, and self-acceptance (though results do not always replicate across samples), and loyalty to family, community, and other groups has been found to correlate positively with happiness [22–24]. However, work that directly addresses the link between moral domains and emotional well-being is limited, and often focuses on moral violations rather than positive moral acts, suggesting a need for further research [e.g., 25].

Second, self-determination theory argues that behaviors can only satisfy a psychological need if they are intrinsically motivated—that is, motivated by the perceived value of the activity itself rather than its instrumental utility. They must also be pursued autonomously (i.e., willingly and volitionally) [17]. A second indicator that morality is a need, then, is whether individuals perform morally motivated behaviors willingly and without external coercion.

Moral principles are ideals that a person considers to be intrinsically right or wrong, and thus intrinsic value arguably characterizes any morally-motivated action [26]. Autonomy, however, could vary across behaviors and circumstances. Prentice and colleagues [7] hypothesize that behaviors associated with “oppressive moral standards”—a category in which they seem to include moral concerns related to loyalty, authority, and sanctity—may be less autonomous and so might yield little to no benefit [20]. However, we argue that any moral concern can be experienced as oppressive or positive, and consequently vary in how autonomously it is enacted. Consider, for instance, the burdens experienced by those who care for children or sick relatives [27, 28]. Here, a genuine commitment to caring for others coexists with the daily realities of difficult work, which means that caregiving behaviors might alternate between being performed willingly and out of a sense of obligation. Likewise, moral standards tied to loyalty, authority, and sanctity-focused morality can be experienced as oppressive—as when people obey to avoid punishment—or performed willingly—as when people cooperate with police authority out of respect for police and the standards they uphold [29]. In our view, fluctuations in autonomy are not inherent in any particular moral principle: they can and do occur for behaviors that correspond to many moral ideals. While it is possible that certain moral values are more likely to prompt autonomous action, ultimately whether any given moral behavior is performed willingly is an empirical question.

Third, the case for morality as a need would be strengthened if we could establish a theoretically consistent account of the causal dynamics among moral behavior, moral need satisfaction, and well-being, ideally using an experimental design [18]. One possibility is that moral behavior encourages individuals to generate positive moral self-appraisals, which in turn boost positive emotions and well-being. Previously research has found support for this idea in the context of prosocial behavior. As noted above, Prentice and colleagues [7] demonstrated that prosocial acts correlated positively with moral self-appraisals, and that moral self-appraisals predicted psychological thriving. Miles and Upenieks [8] showed much the same in an experimental setting, but used positive emotions in place of psychological thriving. In related work, Martela and Ryan [30] found that anonymous good deeds can increase *beneficence*—the sense that one’s actions make the world or the lives of others better—and that beneficence satisfaction, in turn, was associated with higher well-being even when controlling for ACR needs [5]. However, none of these studies formally tested the implied mediation pathway (with the exception of Miles of Upenieks [8]). Further, these studies focused on prosocial acts and thus provide no insight into the possible emotional consequences of engaging in other types of morally motivated actions. It thus remains an open question whether moral self-appraisals mediate the relationship between moral acts (generally) and positive emotions.

This study advances research on morality as a basic psychological need by asking whether moral behavior satisfies the three criteria discussed above. Using an experimental design, a large online sample, and a well-validated behavioral recall paradigm [31], we examine whether: 1) behaviors motivated by moral principles of care, fairness, loyalty, authority, and sanctity can be enacted autonomously—a necessary precondition for psychological need fulfillment; 2) behaviors motivated by any of these moral principles produce positive emotions; and 3) the effects of moral recall on positive emotions will be mediated by moral self-appraisals.

## Materials and methods

Study procedures were approved by the research ethics board at the University of Toronto (protocol number 36829). All study participants gave written consent—i.e., they were shown the consent form and had to click a link to provide consent and begin the survey. Study materials (including data codebook and code to reproduce analyses) are available at https://osf.io/udphs/. We chose an exploratory rather than pre-registered/confirmatory approach given the lack of existing work linking different types of moral behaviors to emotions that could be used to guide our design decisions.

Some of the data used for this study were first reported in Miles and Upenieks [8]. In that paper, only three of the seven experimental conditions were used, as the rest were not relevant to the paper’s argument. The current study uses all seven conditions. See S1 Appendix for a detailed discussion of the differences in how data were coded and analyzed in the two studies.

### Participants and data

We conducted a power analysis to determine how many respondents to recruit. We originally anticipated addressing our work to the prosocial spending literature rather than to the psychological needs literature, so we selected studies for our power analysis that examined prosocial spending using recall tasks. We calculated seven effect sizes from four published articles, which had a median effect size of *d* = 0.42 [32–35]. To balance caution and cost, we used *d* = 0.30 in our power calculations and found that we needed N = 234 per condition to obtain 90% power. We therefore tried to sample 2000 respondents, which would give us approximately 285 respondents per condition (the seven conditions are described below). The surplus respondents were meant to safeguard statistical power in the (likely) event that some responses ended up being unusable.

We recruited N = 2024 respondents from the United States using Amazon’s Mechanical Turk (AMT). AMT is an online crowdsourcing platform subject to several real and potential limitations, including producing non-representative samples, moderately high levels of participation by ineligible respondents, and low attention among some respondents [36, 37]. However, when vetted for low quality data AMT samples have repeatedly been shown to produce experimental effects comparable to those obtained using offline samples, even among respondents who are familiar with study protocols [38, 39]. We describe our procedures for removing low quality responses below.

All data were collected before any analyses were performed. We removed respondents for the following reasons, and in the order shown: those who wrote nothing during the experimental task (N = 7), those who spent less than one second on average answering each item (N = 4; see [40]); those who provided nonsensical or off-topic responses during the experimental task as judged by three independent, crowdsourced raters (also from Amazon’s Mechanical Turk; N = 206, see S2 Appendix for details), and those who showed a problematic pattern of responding (N = 6). Problematic responding was defined as writing very few words during the experimental task (in the 1^st^ percentile) while also having an experimental response time that was either very short or very long (below the 1^st^ percentile or above the 99^th^ percentile). Respondents were also removed if they took a very long time between the start of the experimental task and reporting their emotions (above the 99^th^ percentile; N = 18). This pattern could suggest that respondents were interrupted, which could have disrupted the effect of the experimental prompt. Excluded responses were not systematically related to key study variables, suggesting that their exclusion is unlikely to bias results but might improve the efficiency of estimates. The final sample consisted of N = 1783 respondents.

### Study procedures

At the start of the study, respondents reported their current level of positive affect and then completed a number of preliminary items, including the Moral Foundations Questionnaire (MFQ30). They were then assigned to one of seven experimental conditions (between-subjects): care, fairness, loyalty, authority, purity, self-indulgent purchase, or routine acts. We describe the assignment process here and give full details on each condition below. Assignment to conditions was semi-random. The “semi” randomness arose from the fact that those with high MFQ30 scores for loyalty, authority, or sanctity were disproportionately assigned to the loyalty, authority, or sanctity conditions to increase the chance that the experimental prompts would be meaningful to them. The probabilities we used to make these assignments were based on simulations of the likely sample sizes in each condition given the anticipated distribution of MFQ30 scores in our sample (with those distributions taken from our prior work using Amazon’s Mechanical Turk). Consequently, randomization was correlated with MFQ30 scores, so we controlled for MFQ scores in all analyses. With these controls in the model, assignment to experimental conditions was fully random. Full details on the randomization procedure can be found in S8 Appendix.

As noted, the benefit of this assignment strategy is that respondents received experimental prompts that were likely meaningful to them. The caveat is that it changes how the experimental estimates can be interpreted. Estimates in the loyalty, authority, and sanctity conditions, for instance, cannot be seen as the average effects the experimental procedure would have on anyone, but only the effects it would have on those who value loyalty, authority, and sanctity. This is similar to what is known in the counterfactual causality literature as the average treatment effect for the treated [41]. Conceptually, this corresponds to the question of whether people benefit emotionally when they perform acts that they personally view as moral, which is precisely what we need to determine before we begin testing the morality-as-a-need hypothesis. Arguably, it is also more interesting than the question that our design precludes, which is whether people benefit emotionally when they perform acts that they do not consider to be particularly moral.

Given the difficulty in pre-determining behaviors that would reliably evoke different moral domains for all respondents, we opted to use a recall task so that respondents could select behaviors that they personally viewed as expressing a particular type of morality. Although recalling is not the same as performing behavior, recall tasks have been shown to evoke the emotions associated with original events and to return effect sizes comparable to actual behavior [31, 42].

In each experimental condition, respondents were asked to report in writing a recent time when they had intentionally performed a particular type of behavior, with the type of behavior differing across conditions. Prompts for morality conditions were as follows: “Think back to a recent time that you intentionally” … (**Care**) **“**helped or cared for someone else”, (**Fairness**) “acted honestly or fairly”, (**Loyalty**) “showed loyalty to your nation or another group you belong to”, (**Authority**) “supported or showed respect for a person in a position of authority over you”, (**Sanctity**) “did something to cleanse or strengthen your inner self”. Another condition asked respondents to recall a time when they “bought something for yourself that you really wanted,” and was intended as a positive but (relatively) morally neutral comparison that could be used to help distinguish positive *moral* effects from general positivity. A final condition asked respondents to recount a recent trip to the grocery store and was meant to evoke the affect associated with routine behaviors. In each condition, respondents were encouraged to “describe this experience as vividly and in as much detail as possible,” and were provided with a set of questions to help them create a richer description. These questions were: “What did you do? Why? Who was there? How did they respond? How did you feel?” Per condition sample sizes were N_control_ = 258, N_self-indulgent_ = 277, N_care_ = 250, N_fairness_ = 245, N_loyalty_ = 233, N_authority_ = 245, N_sanctity_ = 275.

After describing their behavior, respondents reported their current affect and then completed measures of moral self-appraisals and the satisfaction of basic psychological needs for autonomy, competence, and relatedness.

Respondents from the routine acts condition were used as controls, though one might ask why we did not instead use respondents from the self-indulgent purchase condition. Theoretically, establishing a basic psychological need does not require that positive effects be stronger than alternative routes to achieving positive outcomes, only that it has positive effects. Given that this is (to our knowledge) the first (exploratory) test of the morality-as-a-need claim that uses multiple types of morality, we opted to use a simpler standard—any difference above a neutral emotional set-point. We realize that some scholars will view this as an uninteresting comparison, but we note that what is “interesting” is highly subjective and does not always map onto what is theoretically informative. That said, we agree that the relative strength of moral vs. self-indulgent acts is interesting, and so present relevant information in supplemental material (see S6 Appendix).

### Measures

#### Experimental condition

Experimental conditions were coded using dichotomous indicators, with the routine acts condition as the reference category.

#### Positive affect

Positive affect was measured using a modified form of the Positive and Negative Affect Scale [see 32, 43, 44]. The same items were administered at the start of the study and immediately following the recall task and rated on a scale ranging from 0 = “not at all” to 8 = “extremely”. Items included excited, alert, active, happy, pleased, proud, elevated, grateful, peaceful, and content (*α*_pre_ = 0.93; *α*_post_ = 0.94).

#### Basic psychological need satisfaction

Autonomy refers to actions that are undertaken willingly and without controlling internal or external pressures [17]. We accordingly asked respondents how much they wanted to perform the behavior they reported during the recall task, and the extent to which they felt obligated to do so (reverse coded). These items did not correlate highly and were kept separate in analyses. Competence is concerned with feeling effective. We measured competence by first asking respondents to think back to the behavior they had reported and then describe what they hoped to accomplish by acting as they did. We then asked the extent to which they thought their behavior accomplished that goal. Responses to the latter question were used as our measure of competence. Relatedness refers to feeling connected to others. We measured relatedness by asking the extent to which respondents hoped that their actions would build or strengthen their relationship with a friend, loved one, or another person, and by asking the extent to which their actions actually helped them feel more socially or emotionally connected to any of those individuals. These two items were averaged to form a scale (*α* = 0.89). Items for all need satisfaction variables were measured using a response scale ranging from 0 = “Not at all” to 8 = “Very much”.

Researchers commonly use the validated scale of Chen and colleagues to measure basic psychological need satisfaction [45]. A post hoc analysis of our measures indicates that our items are similar to items in the Chen et al. scale, though our items are adapted to the behaviors used in this study. They also correspond to the theoretical definitions of autonomy, competence, and relatedness, suggesting that they are likely a valid operationalization of the underlying constructs. See S3 Appendix for a fuller discussion on the validity of these measures.

#### Moral self-appraisals

We asked respondents to think back to the behavior they had described during the recall task and then—based only on how they acted—rate themselves on number of traits. Response options ranged from 0 = “not at all” to 8 = “very much”. Responses for moral, upright, principled, and good were averaged to form a scale that captures moral self-appraisals without reference to any particular type of moral content (e.g., without referencing caring, authority-focused, or other forms of morality specifically, *α* = 0.89). This allowed us to use the same moral self-appraisal measure across all morality conditions, though as Miles and Upenieks [8] note, a generic measure might not capture moral self-appraisals as strongly as measures that reflect particular types of moral content.

#### Controls

All analyses controlled for MFQ30 scores as described previously. Analyses also included a measure of initial affect. This variable is highly correlated with the outcome (post-experimental affect) but not with the experimental conditions, so including it improved the efficiency of the estimates.

Respondents wrote comparable amounts in all conditions, so we did not control for the length of written responses. Time spent writing differed across conditions, with those in the loyalty and authority conditions spending roughly a minute longer on average than those in the control condition. However, time spent on the recall task had no effect on levels of positive affect and so was excluded from analyses.

### Analyses

To increase confidence in the adequacy of our study design to test morality effects, we began by assessing how well our experimental prompts captured the intended moral domains. Next, we determined the extent to which each experimental condition produced feelings of autonomy—a necessary pre-condition for psychological need satisfaction. We then tested whether recalling different types of moral behaviors produced positive emotions, as implied by the claim that morality is a basic psychological need.

To further test the morality-as-a-need hypothesis, we next examined whether observed effects are mediated by moral self-appraisals. We demonstrated mediation using indirect effects—in this case, the indirect effect of recalling moral behavior on positive emotions through moral self-appraisals. As Hayes [46] notes, indirect effects avoid several problems with the classic Baron and Kenny [47] approach to mediation, notably that a non-significant unmediated effect does not necessarily imply that there are no significant mediation pathways (e.g., a non-significant effect might results from two mediation pathways of equal magnitudes but opposite signs; see [46]). Because the sampling distributions of indirect effects need not be normal, we used bootstrapping (5000 replications) and 95% confidence intervals to determine which mediation pathways were statistically significant (using α = 0.05; Hayes, 2018). We also controlled for ACR need satisfaction to isolate mediation through moral self-appraisals from mediation through these alternate pathways.

We used linear models with heteroskedasticity robust standard errors for regression analyses. All non-dichotomous variables were standardized prior to analyses. Consequently, estimates for experimental conditions are standardized mean differences very similar to Cohen’s *d*—the difference is that Cohen’s *d* uses a group-size weighted average of the within-group standard deviations of the two groups being compared (e.g., care condition vs. neutral control condition), while *y*-standardizing uses the total variation (i.e., both within and between group variation) across all experimental groups in the model. In practice, both approaches often yield very similar results. Missing data were adjusted for using full information maximum likelihood [48], and we used *α* = 0.05 as the threshold for statistical significance. 95% confidence intervals for results are shown in brackets. Sample descriptive statistics are given in Tables S4.1 and S4.2 of S4 Appendix, and full analysis results are shown in Tables S4.3-S4.5 of S4 Appendix.

Analyses were performed using R version 4.1.1 [49]. We also used the following packages: tidyverse, lavaan, dotwhisker, rio, psych, robustHD, ngram, lmtest, VGAM, lm.beta, ggsci [50–60].

## Results

### Validating experimental prompts

To ensure that our prompts captured the intended moral domains, we asked crowdsourced coders to assess whether responses from the recall task expressed each type of morality [61]. Coders were given a list of themes (with descriptions) and asked to assess whether those themes were present in each response. The themes were the moral categories of care, fairness, loyalty, authority, and sanctity, as well as several themes suggested by our preliminary review of the data—self-indulgence, guilt, self-improvement, self-therapy, and whether an experience had a positive and/or negative tone. Each response was rated by three coders. See S2 Appendix for details on how coders were selected, and for the theme descriptions we provided.

**Fig 1:**
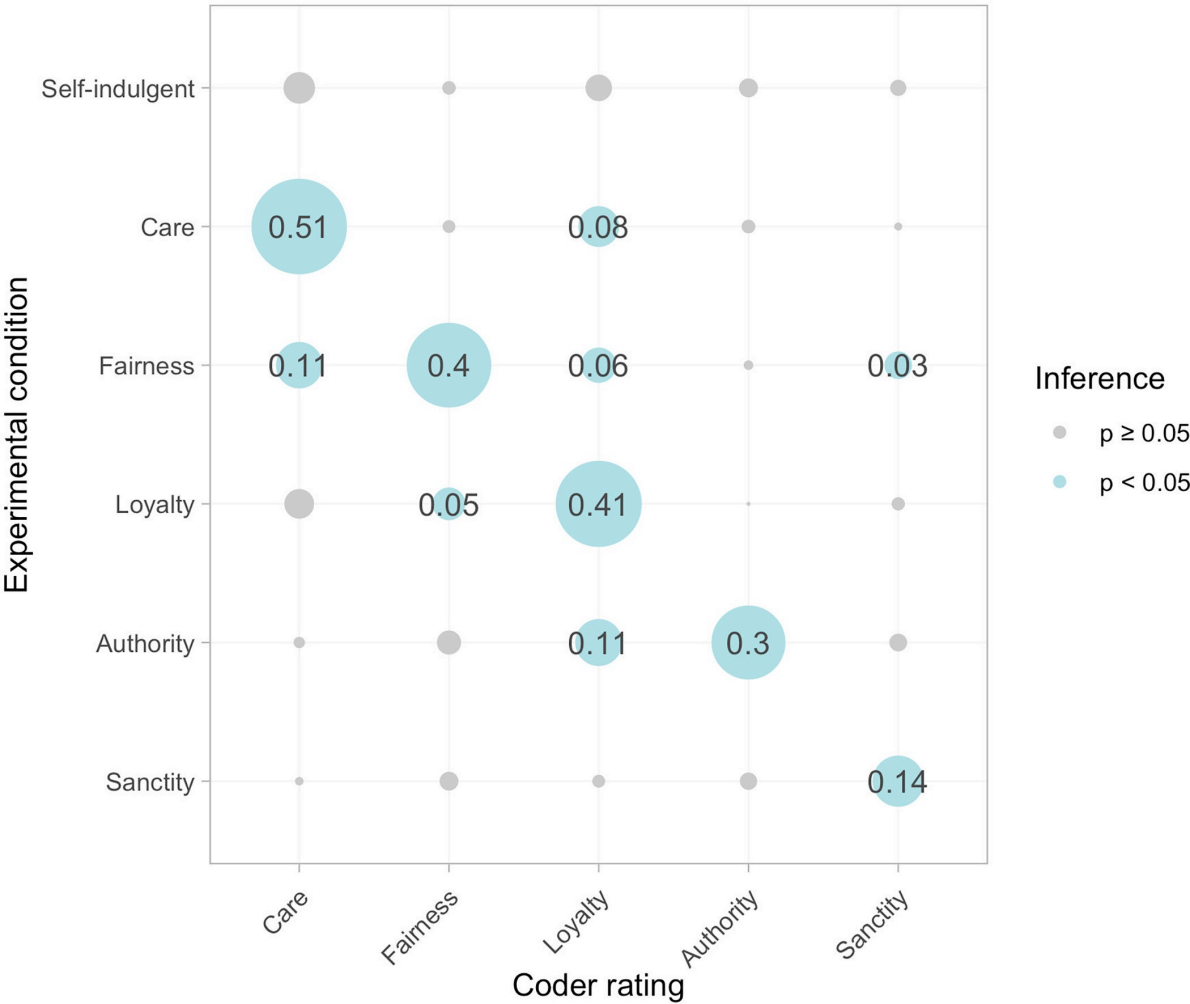
Averaged coder ratings of the moral content present in responses from each experimental condition. Numbers are unstandardized coefficients from a linear regression of averaged coder ratings of the moral content present in responses on indicators for experimental conditions (reference = routine acts control condition). All models used heteroskedasticity-robust standard errors. Missing data were adjusted for using full-information maximum likelihood.

To assess whether our prompts successfully elicited the moral domains we intended, we averaged coder ratings for each response and linearly regressed them on indicators for the moral and self-indulgent experimental conditions (reference = routine behavior). Fig 1 shows a bubble diagram of the coefficients from these models. In each case the relevant moral prompt was by far the strongest predictor of content ratings while other prompts had little to no effect. For instance, coders were more likely to identify “care” content in responses written by those in the care condition than in responses from other conditions (*b*_care_ = 0.51 vs. coefficients of 0.11 or lower for other conditions, 95% CI = [0.44, 0.58], *p* < 0.001). Prompts also accessed the intended domains cleanly. For example, coders rarely reported finding fairness, loyalty, authority, or sanctity in responses from the care condition; finding care, fairness, loyalty or authority in responses from the sanctity condition, and so on.

However, all prompts were not equally powerful in eliciting the associated moral behaviors. Coefficients were largest in the care, fairness, and loyalty conditions, and smaller in the authority and especially sanctity conditions. This suggests that it was easier for study participants to recall—or possibly for coders to identify—behaviors related to care, fairness, and loyalty than behaviors expressing respect for authority or sanctity. Practically, this means that we can have greater confidence that any effects in the care, fairness, and loyalty conditions are attributable to the moral content of recalled behaviors rather than to other, unmeasured features of those behaviors.

### Assessing autonomy need fulfillment

**Fig 2:**
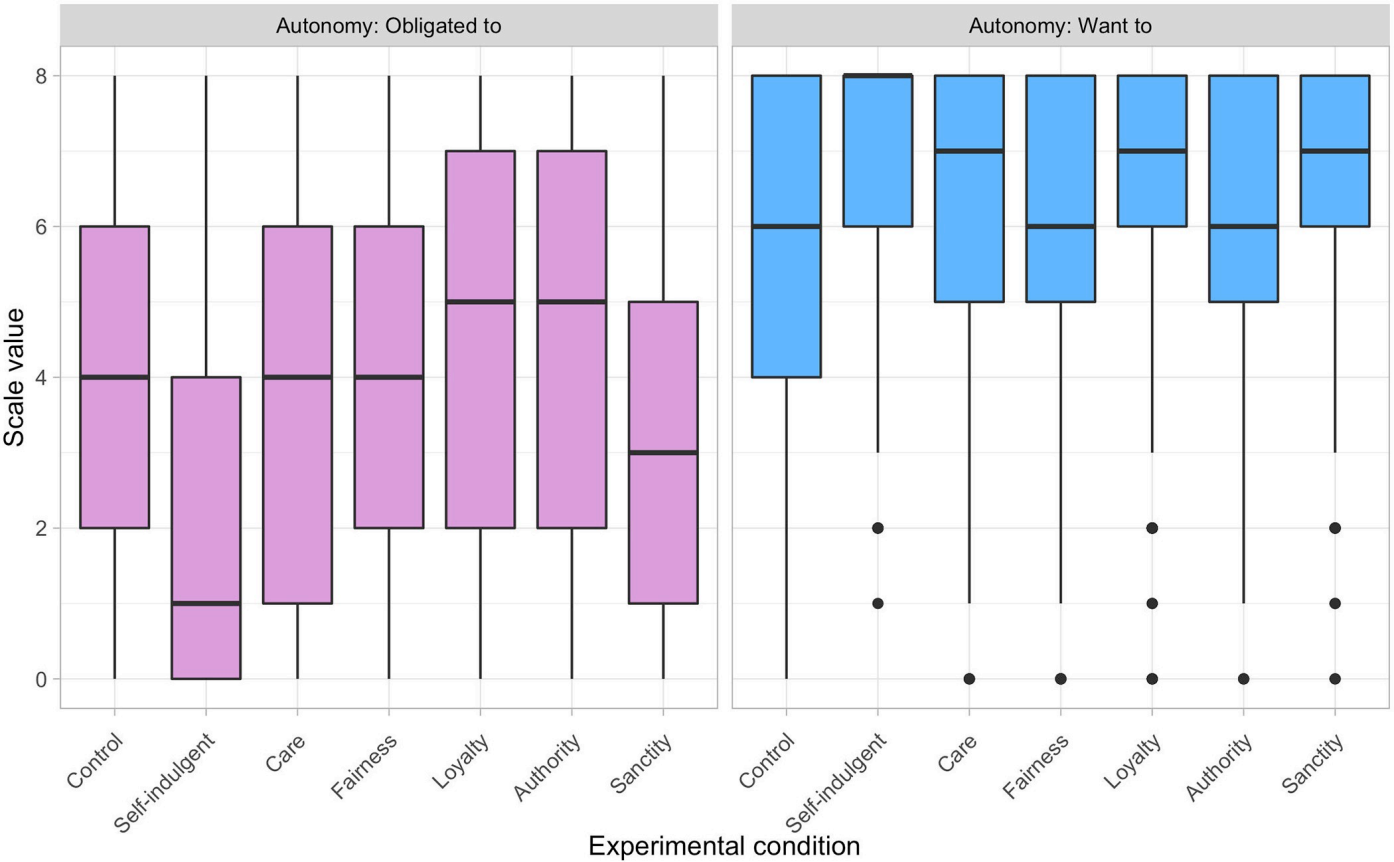
Boxplots of two measures of autonomy, by experimental condition. Autonomy measures capture reported sense of obligation and desire to perform recalled behaviors.

Prentice and colleagues [7] speculated that only certain moral principles are likely to be enacted autonomously, and hence only certain types of moral behavior can satisfy a need for morality. We tested this by examining how well autonomy needs were fulfilled for those recalling different types of moral acts. Fig 2 presents the results for our two measures of autonomy. Obligation—a lack of autonomy—varied widely across all conditions, but medians were close to the scale midpoint in all but the self-indulgent condition. This indicates that respondents generally felt some internal pressure to perform the moral behaviors they reported, but that this pressure was similar for all types of moral acts. Further, the levels reported in each of the morality conditions were roughly parallel to those reported in the routine acts control condition. This suggests that the sense of obligation experienced by those performing moral acts was relatively mundane.

We measured the positive side of autonomy by asking respondents to indicate how much they wanted to perform the behaviors they reported. Responses varied in all conditions, but in every case median values were well into the upper end of the scale, indicating that respondents were generally positively motivated to engage in the moral acts they reported (using Wilcoxon signed rank tests, all medians were significantly above the scale midpoint, all *p*’s <0.001). Again, the striking feature is how similar these values are across morality conditions. Fig 2 thus provides little support for the claim that only some moral principles are likely to motivate autonomous behavior. Of more immediate importance, these results satisfy our first criterion by suggesting that the moral behaviors recalled in our study were performed with high (but not complete) autonomy, and that they therefore could plausibly satisfy basic psychological needs.

### Moral behavior recall, moral self-appraisals, and positive emotions

**Fig 3:**
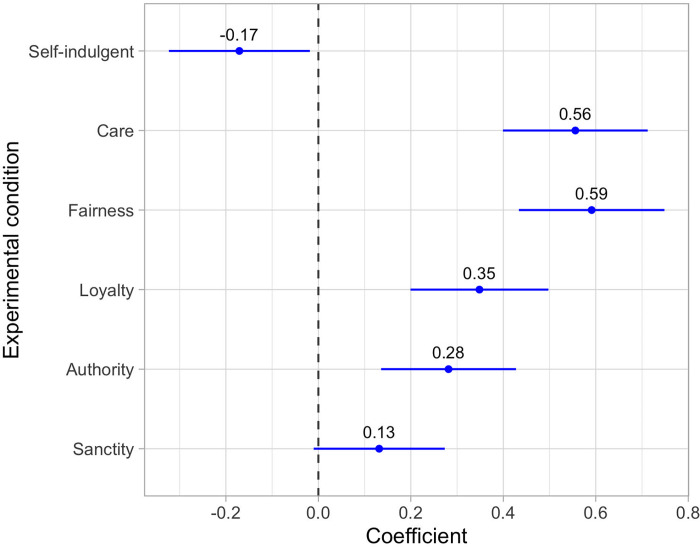
Experimental effects of recalling different types of moral behavior on moral self-appraisals. All models are linear regressions, used heteroskedasticity-robust standard errors, and adjusted for missing data using full information maximum likelihood. Reported effects are in standard deviations of the outcome. Lines represent 95% confidence intervals. The reference category is the routine acts control condition. These results are consistent with the claim that many types of moral behavior can satisfy a psychological need for morality. The small, non-significant effect of sanctity behaviors could temper this claim to the extent it suggests that some morally motivated acts do not generate positive moral self-appraisals. However, given that Fig 1 indicates that the behaviors reported in the sanctity condition had a relatively low level of correspondence to the underlying moral construct, it is also possible that our experimental prompt did not adequately capture sanctity-based morality. If true, then behaviors motivated by sanctity could produce stronger moral self-appraisals under a more appropriate operationalization of sanctity-related moral principles.

According to the theory presented above, satisfying a need for morality first requires seeing oneself as acting morally—that is, a positive moral self-appraisal. Fig 3 demonstrates that respondents in the care, fairness, loyalty, and authority conditions saw themselves as more moral relative to controls. This perception was strongest for those recalling caring or fair actions, who reported moral self-appraisals that were 0.56 and 0.59 standard deviations (SD) greater, respectively, than those in the control condition (*b*_care_ = 0.56 [0.40, 0.71], *p* < 0.001; *b*_fair_ = 0.59 [0.43, 0.75], *p* < 0.001). Effects were smaller but still substantial for those in the loyalty and authority conditions (*b*_loyal_ = 0.35 [0.20, 0.50], *p* < 0.001; *b*_auth_ = 0.31 [0.14, 0.43], *p* < 0.001), but relatively weak and non-significant for those recalling acts of sanctity (*b*_sanc_ = 0.13 [-0.01, 0.27], *p* = 0.069). Notably, recalling a self-indulgent purchase *lowered* moral self-appraisals by about 0.17 SD, suggesting that moral self-appraisals are not just capturing the general positivity of a behavior (*b*_self_ = -0.17 [-0.32, -0.02], *p* = 0.028).

**Fig 4:**
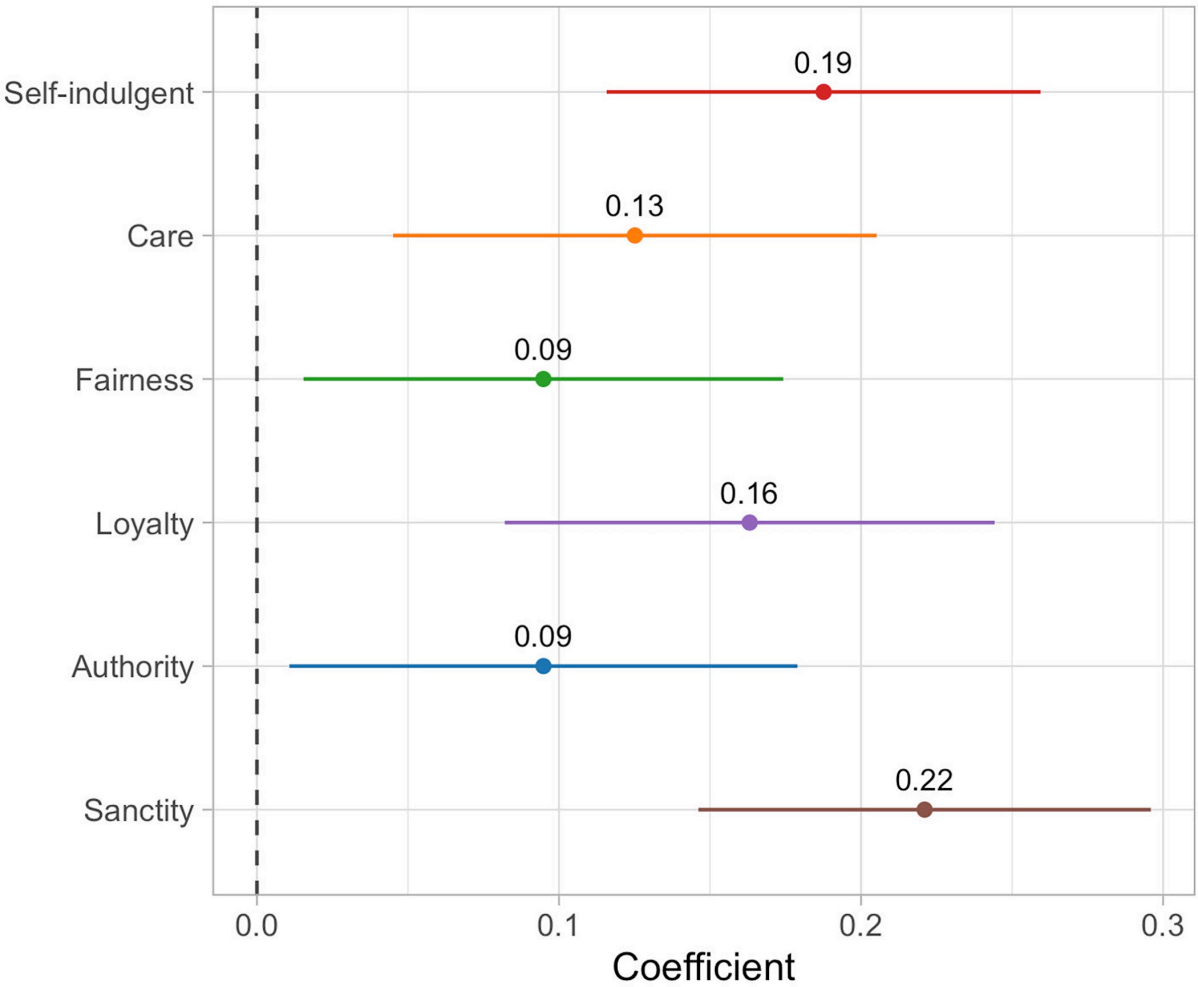
Experimental effects of recalling different types of moral behavior on positive emotion. All models are linear regressions, used heteroskedasticity-robust standard errors, and adjusted for missing data using full information maximum likelihood. Reported effects are in standard deviations of the outcome. Lines represent 95% confidence intervals. The reference category is the routine acts control condition.

Fig 4 shows the effect of recalling different types of moral behavior on positive emotions. All morality conditions and the self-indulgent condition increased positive emotions relative to routine acts controls. The strongest effect was for sanctity, which predicted positive emotions that averaged 0.22 SD above those reported by controls (*b*_sanc_ = 0.22 [0.15, 0.30], *p* < 0.001). Recalling caring, fair, or authority-related acts increased positive emotions by about 0.10 SD, while recalling loyal acts increased positive emotions by 0.16 SD, roughly equivalent to the effect of recalling a self-indulgent purchase (*b*_care_ = 0.13 [0.05, 0.21], *p* = 0.002; *b*_fair_ = 0.10 [0.02, 0.17], *p* = 0.019; *b*_auth_ = 0.10 [0.01, 0.18], *p* = 0.027; *b*_loyal_ = 0.16 [0.08, 0.24], *p* < 0.001; *b*_self_ = 0.19 [0.12, 0.26], *p* < 0.001). Our second criterion for assessing whether morality might plausibly be a basic psychological need is thus confirmed for all experimental conditions.

We can gain a better sense of the magnitude of these effects by comparing them to effects found in past work. As noted previously, standardized estimates from our analyses are roughly equivalent to estimates of Cohen’s *d*, allowing us to compare them to published results directly (estimates in Fig 4 differ by no more than 4% from the equivalent estimates of Cohen’s *d*, and are identical when both are rounded to two decimal places as presented here). A recent meta-analysis of prosocial acts on positive emotions found an effect of *d* = 0.28 [1], though this figure is likely inflated by small sample bias. A small sample adjusted meta-analysis of positive psychology interventions on well-being (including but not limited to acts of kindness) found an effect of *d* = 0.20 (*r* = 0.10) which was only one third as large as the effect found in a prior meta-analysis of the same studies [62]. If we assume that appropriate adjustments for small-sample bias would lead to similar reduction in the prosocial meta-analytic estimate, then we should expect an effect of about *d* = 0.09. This is slightly larger than the *d* = 0.06 found in a large, pre-registered replication of a prosocial spending recall experiment [63]. Taken together, this work suggests that positive/prosocial interventions produce effects somewhere between *d* = 0.06 and *d* = 0.20. Our effects largely fit within this range, running from 0.10 for fairness and authority to 0.22 for sanctity (see S5 Appendix for a further discussion of these effect sizes; see S6 Appendix for a comparison to the self-indulgent purchase condition).

The experimental effects shown in Figs 3 and 4 demonstrate that recalling moral acts both encouraged positive moral self-appraisals and increased positive emotions. The next step is determining whether these effects are linked along a mediation pathway: in particular, that moral recall produced positive emotions because it first generated moral self-appraisals. We tested this by adding moral self-appraisals and ACR needs into the model linking experimental conditions to positive emotions, and then calculating the indirect effects of each condition on positive emotion through moral self-appraisals and each ACR need. In contrast to the analyses presented in Figs 3 and 4, these indirect effects cannot be interpreted causally because the mediators—moral self-appraisals and ACR needs—were not experimentally manipulated. Consequently, the presence of indirect effects should be seen as suggestive rather than definitive evidence of mediation.

**Fig 5:**
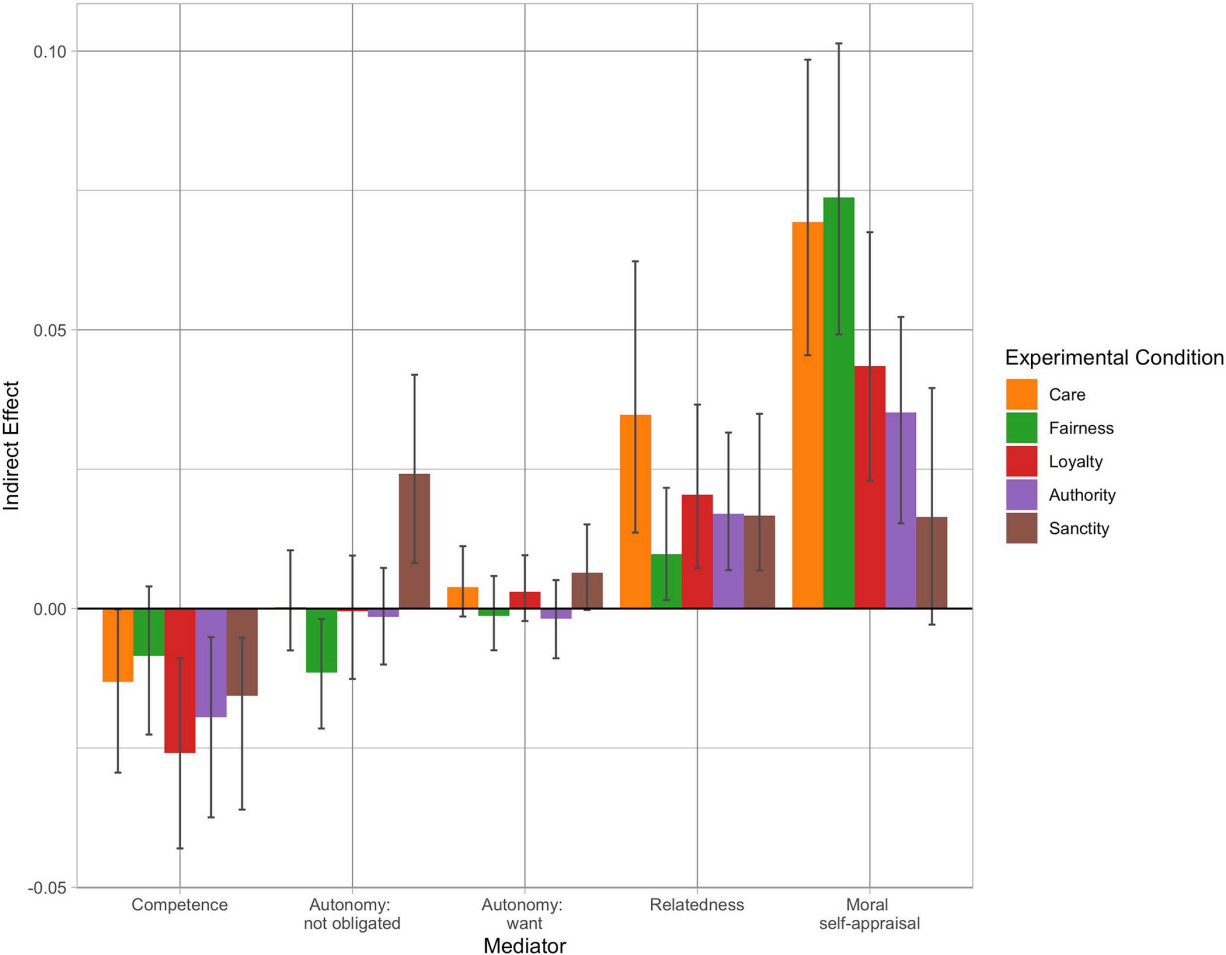
Indirect effects of recalling moral behavior on positive affect. Effects are shown using moral self-appraisals and ACR need satisfaction as mediators. The reference category is the routine acts control condition. Error bars display bootstrapped 95% confidence intervals.

Estimated indirect effects are shown in Fig 5 along with bootstrapped 95% confidence intervals (see Table S4.6 in S4 Appendix for results in numeric form). Fig 5 shows that recalling moral behavior generally prompted no differences in positive emotion through autonomy need satisfaction. Surprisingly, competence had small *negative* indirect effects on positive emotion for those recalling acts of care, loyalty, respect for authority, or sanctity. This is because these acts reduced feelings of competence relative to controls, a fact which likely reflects the difficulty of accomplishing morally motivated goals compared to routine acts. The *positive* effects of moral recall on positive emotions thus cannot be explained by the satisfaction of competence or autonomy needs.

In contrast, indirect effects through both relatedness and moral self-appraisals were positive and statistically significant in almost every condition (the exception being the non-significant indirect effect of recalling sanctity acts through moral self-appraisals). Indirect effects through moral self-appraisals were by far the largest of any indirect effects calculated. It is reasonable to wonder if this strong performance simply reflects the superior psychometric properties of the moral self-appraisals measure, which was measured with four items compared to one or two items for the ACR needs measures. However, sensitivity analyses using several two-item versions of the moral self-appraisals measure suggest that this is not the case (see S7 Appendix for details). More to the point, the large indirect effects through moral self-appraisals in this study does not necessarily suggest that moral self-appraisals will always be stronger mediators than relatedness (or competence or autonomy) need satisfaction. We therefore caution against emphasizing the strength of moral recall effects through moral self-appraisal effect relative to the effects through ACR needs. Rather, the key takeaway is that our results support our third criterion: that moral acts produce positive emotions because they allow people to view themselves as more moral.

## Discussion

Psychologists have explained the salubrious effects of prosocial behavior as the result of fulfilling basic psychological needs for autonomy, relatedness, and competence [5], but recent evidence suggests a fourth need may profitably be added to the fold: the need to feel moral [6, 7]. Establishing any construct as a psychological need requires satisfying numerous criteria, and no one study alone can definitively make the case [16, 17]. We build on early-stage work by testing three implications of the morality-as-a-need perspective.

First, psychological needs can only be satisfied when behaviors are enacted autonomously. Consistent with this, we found that respondents generally wanted to perform the moral behaviors they recalled. Interestingly, this desire was accompanied by a modest sense of obligation, which could suggest a level of unwillingness as well. However, the levels of obligation in the moral behavior conditions were similar to those in the routine acts control condition, suggesting that any unwillingness was relatively mild. Further, it is possible that a minor sense of obligation might be compatible with full autonomy. Behaviors that may at first seem obligatory (e.g., chatting with a neighbor, helping a friend out) may over time become valuable and allow people to fulfill larger, autonomously chosen goals, even if those behaviors are not always pleasant in the moment. Determining the extent to which obligation can be compatible with autonomy is an interesting subject for future research.

After establishing that moral behaviors could be enacted autonomously, we tested the claim that morality is a need by evaluating one of its major theoretical implications: that positive emotions should follow not just prosocial acts, but any behavior that a person regards as moral. Our results support this prediction by showing that those who recalled behaviors associated with care, fairness, loyalty, authority, and sanctity-based morality experienced more positive emotions than those who recalled routine daily acts. These effects were small, but similar to those found in past research on the positive emotional effects of prosocial behavior and other positive psychology activities [1, 62, 63]. Finally, we examined whether moral self-appraisals could explain the observed effects. We found that moral recall effects were mediated by moral self-appraisals, as well as the satisfaction of a basic psychological need for relatedness. While our analyses are consistent with prior theory about how moral acts generate positive emotions, they could not establish whether these mediation pathways are causal. We return to this point below.

Prentice and colleagues [7] hypothesized that some moral domains are more conducive to moral need satisfaction than others because they are more readily enacted in an autonomous fashion. In contrast, we found little difference in autonomy need satisfaction across different moral behaviors. We did, however, find that moral behaviors predicted moral self-appraisals to varying degrees, with the strongest relationships arising for care and fairness, just as Prentice and colleagues suggested. This may be because behaviors motivated by care and fairness more easily give rise to moral self-appraisals as they claim. An alternative is that this discrepancy reflects features of our study design, such as the experimental prompts we used. This seems a particularly likely explanation for the small effect of sanctity on moral self-appraisals in light of the important place sanctity-related issues (such as sexual behavior) occupy in many people’s moral and political worldviews [20, 64]. Another possibility is that the effects of care and fairness morality were stronger because they are more widely perceived as moral principles. Consequently, the weaker effects of the loyalty, authority, and possibly sanctity conditions could reflect the fact that a lower proportion of individuals in these conditions would have regarded the acts they were describing as moral. This could have occurred even with our MFQ30-informed randomization because the probabilistic nature of the process did not ensure that *all* respondents in the loyalty, authority, and sanctity conditions regarded these principles as morally-binding. In our view, the claim that morality is a basic psychological need seems more plausible if morality effects are not restricted to a narrow subset of moral concerns. Thus, eliminating design issues as a possible explanation for why different moral behaviors predict different levels of moral self-appraisal is an important task for future research.

Our findings are consistent with the hypothesis that morality is a basic psychological need, but substantial work is needed to fully establish this claim. One crucial task is to more firmly establish whether moral self-appraisals mediate the effect of moral recall (or acts) on positive emotions. While our study provides evidence against certain alternatives—such as that indirect effects through moral self-appraisals are completely absent—our design cannot rule out other competing explanations, such as the possibility that positive emotions caused people to rate themselves as more moral, rather than the reverse. Future work should therefore use stronger designs such as manipulation-of-mediator approaches to establish whether moral self-appraisals act as mediators, as the moral-need hypothesis implies [65, 66].

Researchers should also replicate the current results. This paper used exploratory approaches given the novelty of the hypotheses, but future work should build on this foundation to pre-register data collection and analysis plans to mitigate possible influences of idiosyncratic researcher decisions. It will also be important to replicate results using offline, preferably nationally representative samples. While previous work replicating experimental effects online gives us some reassurance that our results are sound [38, 39], the fact that samples from Amazon’s Mechanical Turk tend to be more liberal and less cooperative and generous—among other differences—means that the size of our estimated effects might be biased compared to effects calculated from representative data [36, 67]. This might be particularly true in the loyalty, authority, and sanctity conditions as these forms of morality are often more highly valued among conservatives [68].

Beyond exact replications, replication efforts should also use a wider array of morality conditions, including conditions that involve actual (rather than recalled) behavior. Particular attention should be given to establishing whether sanctity-based moral behavior can generate strong moral self-appraisals. The relationship between these two was weak in our study, but this could reflect an inadequate operationalization of the sanctity construct in our experimental design. In particular, we asked respondents what they did to “cleanse or strengthen your inner self” in an effort to elicit behaviors aimed at metaphorical or spiritual purification—the positive, proactive side of sanctity—and to avoid reactive behaviors aimed predominately at protecting the self from moral contamination. However, crowdsourced coding revealed that this prompt seems to have captured experiences of self-therapy and self-improvement rather than moral content per se. Additionally, these themes did not explain the effect of sanctity behavior on positive emotions in supplemental analyses.

Morality also did not explain the positive effects of recalling a self-indulgent purchase. Those in the self-indulgent condition showed lower levels of moral self-appraisals than those recalling a trip to the grocery store, perhaps reflecting a belief among some that either a) grocery shopping has some moral value (e.g., as a fulfillment of a duty), or b) that self-focused behavior is somewhat immoral (e.g., because it is selfish). Supplemental analyses based on crowdsourced coding (available in the study code files) indicate that self-indulgence is seen as more pleasant/positive than recalled behaviors in any other condition, but that it is not seen as particularly self-therapeutic or aimed at self-improvement. Rather, we suspect that respondents saw self-indulgence as positive because indulging oneself is a form of hedonic goal pursuit, which scholars have shown predicts emotional well-being [69].

Our study focused on the positive emotional consequence of moral self-appraisals and the ability of moral behavior to be enacted autonomously, but establishing morality as a need will require testing a range of other criteria [7, 17]. For example, thwarting need satisfaction should have negative effects on well-being, which suggests that future work should determine whether people who are prevented from acting morally suffer negative emotional consequences. Further, both the positive and negative effects of need satisfaction and thwarting should apply universally. Thus, researchers should examine whether these effects appear in diverse samples, such as in people from different countries or cultural groups [18]. Future work could also test the efficacy of moral behavior for producing moral self-appraisals and generating positive emotion in groups that are observed across situations and over time (see S5 Appendix).

Substantial work remains to determine whether morality is a basic psychological need, but in our view this work is worth undertaking. Theoretically, establishing morality as a need would give insight into what it means to be human, and help illuminate the role morality has played in human affairs historically as well as the role it continues to play today [e.g., 70]. Practically, a moral need would mean that individuals can improve their emotional well-being by pursuing a wide range of behaviors, which in turn implies that these emotional benefits are available in many ways to many people. Currently the underlying evidence base is still quite sparse, so we cannot yet reap the theoretical benefits, nor recommend moral action as a reliable means of improving well-being. We instead encourage scholars to continue working on this important topic so these benefits can (potentially) be realized in the future.

## Supporting information

S1 Appendix Comparison with Miles and Upenieks 2021. (DOCX)

S2 Appendix Details on crowdsourced coding. (DOCX)

S3 Appendix Validating the basic psychological needs measures. (DOCX)

S4 Appendix Sample descriptive statistics and full analysis results. (DOCX)

S5 Appendix Effect sizes and morality as a basic psychological need. (DOCX)

S6 Appendix Comparison of estimates for moral recall and self-indulgent purchase conditions. (DOCX)

S7 Appendix Alternate coding of moral self-appraisals. (DOCX)

S8 Appendix Details on randomization procedure. (DOCX)
