# Supplementary material for: Beyond prosociality: Recalling many types of moral behavior produces positive emotion
Source: PLoS One. 2022 Nov 11;17(11):e0277488. doi: 10.1371/journal.pone.0277488 (PMC9651559; doi:10.1371/journal.pone.0277488)
Supplement: S1 Appendix — (DOCX) [file pone.0277488.s001.docx]

**Supporting information 1: Comparison with Miles and Upenieks 2021**

In Miles and Upenieks [1]—hereafter MU—we (Miles and Upenieks) used data from the control, self-indulgent, and care experimental conditions of the current study to test whether moral self-appraisals mediated the effect of recalling a caring act on positive affect. This naturally raises the question: How much overlap is there between MU and the current paper? There are two potential concerns. 1) Are the analyses sufficiently distinct from those in MU to merit publication in a separate article? 2) Where analyses are similar, do the current paper and MU come to different conclusions, which could suggest results are artifacts of researcher decisions in data coding and analysis? This online supplement provides answers to these questions.

*Distinctiveness of Analyses*

Both MU and the current study use many of the same variables (e.g., positive affect), but we developed the analyses independently to reflect the different aims of the two papers. MU was intended as a test of the mechanisms underlying the well-established effects of prosocial (caring) behavior on emotions, while the current paper is meant to establish whether positive emotional effects can arise from any morally motivated behavior, with the mechanism (mediation by moral self-appraisals) a secondary concern. Consequently, most analyses are unique, with two exceptions which we investigate below. Additionally, separate development means that we coded data somewhat differently across studies.

**Sample Size.** We applied a more stringent data exclusion approach in the current study, which led to somewhat smaller sample sizes compared to the MU analysis. In the MU analysis, per condition sample sizes were N_control_ = 273, N_self-indulgent_ = 296, and N_care_ = 269. In the current study, sample sizes for those same conditions are N_control_ = 258, N_self-indulgent_ = 277, and N_care_ = 250.

**Variable Coding.** The measure of positive affect is identical in both MU and the current study, as are the control variables used in analyses. However, the measure of moral self-appraisals differs. In the current study, moral self-appraisals are measured using a 4-item scale that taps generic morality—that is, perceptions of oneself as being moral without reference to any particular type of moral content. This makes it straightforward to compare moral self-appraisals across experimental conditions aimed at eliciting different types of moral content, which is a primary aim of the current study. In contrast, MU measured moral self-appraisals with a single item focused on how helpful a respondent felt that they acted. This approach reflected MU’s focus on prosocial action and kept the analysis consistent with other studies reported in that paper. The key point is that the moral self-appraisals measured in the current paper and in MU are measuring different types of moral self-appraisals and are not directly comparable.

The current analysis also differs in how it controls for basic psychological need satisfaction. In MU, autonomy, competence, and relatedness (ACR) needs were measured with single items so that both ACR needs and moral self-appraisals would be measured using the same number of items. We did this to reduce the possibility that differences in calculated effect sizes reflected differences in measurement reliability rather than differences in the effects of the underlying constructs. In the current study, however, we used all available items for ACR needs to provide maximum control for ACR need satisfaction. Autonomy is measured with two separate items, competence with a single item, and relatedness with a two-item scale.

*How Much do Coding Differences Matter?*

The effects of coding differences between the current study and MU can only be evaluated for the direct effects of the self-indulgent and care experimental conditions on positive affect, as these are the only effects that are estimated in both studies.

Differences in sample size make little difference to the results. The y-standardized estimate for the effect of recalling a self-indulgent purchase (vs. control) is the same in both studies when rounded to two decimal places: β=0.19, with *p*<0.001. Similarly, the estimate for the effect of recalling caring acts is β=0.12 (*p*=0.005) in MU, and β=0.13 (*p*=0.002) in the current study. These results show that while there are some differences in the exact estimates across studies, they are trivially small. This suggests that the results of the current study are not the product of a highly idiosyncratic strategy of data exclusion.

How sensitive are our results in the current paper to our choice of how to control for ACR need satisfaction? Table S1.1 presents the results from a model that compares the estimates from the current paper to a model that is almost identical, except that it uses the same single item measure coding for ACR need satisfaction that we used in MU. The pattern of results is identical, and in most cases differences in the estimates are small enough that they disappear entirely when results are rounded to two decimal places. As before, these results indicate that our findings are robust to small differences in data coding.

**Table S1.1 Comparison of key coefficients from models using the current coding of ACR need variables and the coding used in Miles and Upenieks (2021)**. Compare to Table S4.5 in supporting information S4 appendix. Standard errors are heteroskedasticity-robust. 95% confidence intervals are shown in brackets. Estimates are adjusted for missing data using full information maximum likelihood.

|  | *Current coding* | | | | |  | *MU coding* | | | | |
| --- | --- | --- | --- | --- | --- | --- | --- | --- | --- | --- | --- |
|  | **Est.** | **S.E.** | **p** | **95% CI** | |  | **Est.** | **S.E.** | **p** | **95% CI** | |
| *Experimental Conditions* | | | | | | | | | | | |
| Self-indulgent | 0.18 | (0.04) | <0.001 | [0.10, | 0.25] |  | 0.21 | (0.04) | <0.001 | [0.13, | 0.28] |
| Care | 0.03 | (0.04) | 0.472 | [-0.05, | 0.11] |  | 0.04 | (0.04) | 0.345 | [-0.04, | 0.12] |
| Fairness | 0.03 | (0.04) | 0.418 | [-0.05, | 0.11] |  | 0.03 | (0.04) | 0.514 | [-0.05, | 0.11] |
| Loyalty | 0.12 | (0.04) | 0.002 | [0.05, | 0.20] |  | 0.13 | (0.04) | 0.001 | [0.05, | 0.20] |
| Authority | 0.07 | (0.04) | 0.111 | [-0.02, | 0.15] |  | 0.07 | (0.04) | 0.081 | [-0.01, | 0.15] |
| Sanctity | 0.17 | (0.04) | <0.001 | [0.10, | 0.25] |  | 0.20 | (0.04) | <0.001 | [0.13, | 0.28] |
| *Mediators* | | | | | | | | | | | |
| Moral self-appraisal | 0.12 | (0.02) | <0.001 | [0.09, | 0.16] |  | 0.12 | (0.02) | <0.001 | [0.09, | 0.15] |
| Competence | 0.07 | (0.01) | <0.001 | [0.04, | 0.10] |  | 0.07 | (0.01) | <0.001 | [0.04, | 0.10] |
| Autonomy: not obligated | 0.05 | (0.01) | <0.001 | [0.03, | 0.07] |  | 0.07 | (0.01) | <0.001 | [0.04, | 0.10] |
| Autonomy: want | 0.03 | (0.01) | 0.014 | [0.01, | 0.06] |  | 0.04 | (0.01) | 0.005 | [0.01, | 0.06] |
| Relatedness | 0.04 | (0.01) | 0.001 | [0.02, | 0.07] |  | 0.01 | (0.00) | 0.009 | [0.00, | 0.02] |

In summary, the current paper and MU differ in the theoretical questions they address, and consequently code key variable differently and perform different analyses. In the two cases where results are directly comparable, differences in sample composition and controls did not alter substantive results, suggesting that these results are robust to small variations in data coding decisions.

**S1 References**

1. Miles A, Upenieks L. Moral Self-Appraisals Explain Emotional Rewards of Prosocial Behavior. J Happiness Stud. 2022;23: 1793–1814. doi:10.1007/s10902-021-00434-w
