# Supplementary material for: Beyond prosociality: Recalling many types of moral behavior produces positive emotion
Source: PLoS One. 2022 Nov 11;17(11):e0277488. doi: 10.1371/journal.pone.0277488 (PMC9651559; doi:10.1371/journal.pone.0277488)
Supplement: S2 Appendix — (DOCX) [file pone.0277488.s002.docx]

**Supporting information 2: Details on Crowdsourced Coding**

We coded written responses from the recall task in two stages. First, we recruited workers from Amazon’s Mechanical Turk to determine whether responses complied with task instructions [1]. We asked workers to assess whether each response “describe[s] a person’s behavior”. This measure is a conservative test, as it assesses a basic, reasonably objective level of compliance (reporting a behavior) and not forms of compliance that might be more subjective, such as whether respondents provided on-topic or detailed descriptions. Three coders rated each item. Responses that were judged as non-compliant by two out of three raters were removed from the sample, as described in the Data section of the paper.

Compliant responses were then submitted to a second round of coding. We administered a qualification test to 100 workers from Amazon’s Mechanical Turk. The test asked workers to determine whether particular themes were present in four written responses from our data. The themes were the moral categories of care, fairness, loyalty, authority, and sanctity, as well as several themes suggested by our preliminary review of the data—self-indulgence, guilt, self-improvement, self-therapy, and whether or not an experience had a positive or negative tone. Coders were provided with the following descriptions:

- **Care**: helping others, relieving suffering
- **Fairness**: seeking justice or equality, maintaining the rights of self or others
- **Loyalty**: supporting or showing allegiance to a group such as a nation, club, or family
- **Authority**: supporting or showing respect for a person or organization in an authority position, such as a leader or the government
- **Sanctity**: seeking personal cleanliness or sanctity, such as through self-discipline or spiritual/religious practice
- **Self-indulgence**: doing something for oneself that is enjoyable or pleasurable
- **Guilt**: acting out of a sense of remorse, or to avoid feeling remorse
- **Self-improvement**: effort to improve oneself in some way
- **Self-therapy**: trying to relax, decompress, or cope
- **Positive**: the experience is described as being pleasant or positive in some way
- **Negative**: the experience is described as being unpleasant or negative in some way

Correct answers for the coding qualification test were agreed upon among the three authors. Workers who coded with at least an 86% accuracy were invited to code responses from the recall task for the same themes. Seventeen coders qualified. Responses were coded in batches of 30 (and one batch of 15), with 1905 responses coded in total. Each response was coded by three workers. These ratings were averaged and used in the analyses presented in “Validating Experimental Prompts” section of the paper.

**S2 References**

1. Tosti-Kharas J, Conley C. Coding psychological constructs in text using mechanical turk: A reliable, accurate, and efficient alternative. Front Psychol. 2016;7: 1–9. doi:10.3389/fpsyg.2016.00741
