# Supplementary material for: Beyond prosociality: Recalling many types of moral behavior produces positive emotion
Source: PLoS One. 2022 Nov 11;17(11):e0277488. doi: 10.1371/journal.pone.0277488 (PMC9651559; doi:10.1371/journal.pone.0277488)
Supplement: S3 Appendix — (DOCX) [file pone.0277488.s003.docx]

**Supporting information 3: Validating the Basic Psychological Needs Measures**

Basic psychological need fulfillment is often assess using the Basic Need Satisfaction and Frustration Scales (BNSFS) [1]. However, we did not become aware of this scale until after our research had commenced. By that time, we had already developed and used our own measures of psychological need fulfillment. These measures were formulated after consulting the theoretical descriptions of autonomy, competence, and relatedness, and designed to capture the major thrust of each construct [2].

In what follows, we examine the adequacy of our measures by comparing them to the theoretical descriptions of autonomy, competence, and relatedness found in the literature, and by comparing our measures to the items used in the BNSFS.

*Theoretical Validation*

Autonomy, competence, and relatedness are broad constructs and cannot fully be captured by the 1-2 item measures we used. However, our measures capture the core components of each construct [2 chapter 4].

Autonomy refers to actions that are undertaken willingly and without controlling internal or external pressures. We accordingly asked respondents how much they *wanted* to perform the acts they described, and how *obligated* they felt to perform those acts (reverse coded). Wanting suggests willingness, and a lack of obligation indicates a lack of controlling pressures.

Competence is about feeling effective. Effectiveness implies a goal, so we first asked respondents to think back to the experience they had recalled and to describe what they hoped to accomplish by acting as they did. To assess competence, we then asked how well they thought their behavior accomplished that goal.

Relatedness is about feeling connected to others. We measured relatedness by asking the extent to which respondents’ actions helped them feel more socially or emotionally connected to a friend, loved one, or another person.

*Conceptual Comparison to the BNSFS*

Table S3.1 shows the need satisfaction items from the established BNSFS. Items from our study are shown alongside the items from the BNSFS that we believe they most closely correspond to. Table S3.1 makes it clear that our measures are conceptually quite similar to the items used in the BNSFS. The BNSFS, for instance, measures autonomy by asking about choice and freedom, while our measure asks about obligation, a significant perceived constraint on choice (reversed for analyses). Both the BNSFS and our measures also ask directly whether actions/decisions reflect what a person wants. BNSFS relatedness items ask about feeling connected to caring and important others. Our items similarly assess connections to others, including to (generally) caring and important others like friends and loved ones. BNSFS competence items refer to achieving goals, feeling capable, and completing difficult tasks. Our competence item asks directly about how well a person has achieved a goal, which in turn implies that a person can complete goal-relevant tasks and is generally capable of achieving that goal.

**Table S3.1 Comparison of Items Used to the items in the Basic Need Satisfaction and Frustration Scales**

| **Items from** **BNSFS** | **Items from this study** |
| --- | --- |
| *Autonomy* |  |
| I feel a sense of choice and freedom in the things I undertake. | Think about the experience you described. To what extent did you feel *obligated* act as you did? (reverse coded) |
| I feel that my decisions reflect what I really want. | Think about the experience you described. To what extent did you *want* to act as you did? |
| I feel I have been doing what really interests me |  |
| I feel my choices express who I really am. |  |
| *Relatedness* |  |
| I feel that the people I care about also care about me |  |
| I feel connected with people who care for me, and for whom I care | Think about the experience you described. To what extent did your action(s) help you to feel more socially or emotionally connected to a friend, loved one, or another person?  Think about the experience you described. To what extent did you hope that your action(s) would build or strengthen your relationship with a friend, loved one, or another person? |
| I feel close and connected with other people who are important to me |  |
| I experience a warm feeling with the people I spend time with |  |
| *Competence* |  |
| I feel competent to achieve my goals | To what extent do you think that your behavior accomplished what you intended? |
| I feel capable at what I do |  |
| I feel I can successfully complete difficult tasks |  |
| I feel confident that I can do things well |  |

In addition to highlighting the conceptual similarities between our measures and those used in the BNSFS, Table S3.1 also shows that our measures are tailored to the particular behaviors that we studied. That is, rather than assessing psychological need satisfaction *generally* as the BNSFS does, we assessed the satisfaction afforded by the *particular* behaviors that people reported. This strikes us as a decided advantage. Theoretically, there is little reason to suppose that a single moral act would shift overall evaluations of basic psychological need satisfaction, but there is ample reason to suppose that it would influence feelings of autonomy, competence, and/or relatedness in a particular moment. Our measures thus captured the type of need satisfaction most likely to occur in the context of our study.

**S3 References**

1. Chen B, Vansteenkiste M, Beyers W, Boone L, Deci EL, Van der Kaap-Deeder J, et al. Basic psychological need satisfaction, need frustration, and need strength across four cultures. Motiv Emot. 2015;39: 216–236. doi:10.1007/s11031-014-9450-1

2. Ryan RM, Deci EL. Self-Determination Theory: Basic Psychological Needs in Motivation, Development, and Wellness. New York: Guilford Press; 2018.
