# Supplementary material for: Beyond prosociality: Recalling many types of moral behavior produces positive emotion
Source: PLoS One. 2022 Nov 11;17(11):e0277488. doi: 10.1371/journal.pone.0277488 (PMC9651559; doi:10.1371/journal.pone.0277488)
Supplement: S4 Appendix — (DOCX) [file pone.0277488.s004.docx]

**Supporting information 4: Sample Descriptive Statistics and Full Analysis Results**

*Tables with Descriptive Statistics*

**Table S4.1 Sample description**. N = 1783. Statistics were adjusted for missing data using full information maximum likelihood estimation.

|  | **Mean** | **S.D.** | **Min** | **Max** |
| --- | --- | --- | --- | --- |
| *Experimental Conditions* |  |  |  |  |
| Self-indulgent | 0.16 | --- | 0 | 1 |
| Care | 0.14 | --- | 0 | 1 |
| Fairness | 0.14 | --- | 0 | 1 |
| Loyalty | 0.13 | --- | 0 | 1 |
| Authority | 0.14 | --- | 0 | 1 |
| Sanctity | 0.15 | --- | 0 | 1 |
| Competence | 6.44 | 1.84 | 0 | 8 |
| Autonomy: not obligated | 4.29 | 2.84 | 0 | 8 |
| Autonomy: want | 6.27 | 1.98 | 0 | 8 |
| Relatedness | 3.55 | 2.69 | 0 | 8 |
| Moral self-appraisal | 5.70 | 1.99 | 0 | 8 |
| Positive affect | 4.19 | 1.99 | 0 | 8 |
| Positive affect (pre-study) | 3.90 | 1.87 | 0 | 8 |
| MFQ:Care | 4.62 | 0.86 | 1 | 6 |
| MFQ:Fairness | 4.57 | 0.80 | 1 | 6 |
| MFQ:Loyalty | 3.33 | 1.02 | 1 | 6 |
| MFQ:Authority | 3.65 | 1.00 | 1 | 6 |
| MFQ:Sanctity | 3.32 | 1.30 | 1 | 6 |

**Table S4.2 Descriptive statistics of key study variables, by experimental condition.**

|  | **Control** | | **Self-indulgent** | | **Care** | | **Fairness** | | **Loyalty** | | **Authority** | | **Sanctity** | |
| --- | --- | --- | --- | --- | --- | --- | --- | --- | --- | --- | --- | --- | --- | --- |
|  | **Mean** | **S.D.** | **Mean** | **S.D.** | **Mean** | **S.D.** | **Mean** | **S.D.** | **Mean** | **S.D.** | **Mean** | **S.D.** | **Mean** | **S.D.** |
| Positive affect | 3.86 | 2.01 | 4.20 | 1.94 | 3.56 | 1.92 | 3.52 | 1.83 | 4.52 | 2.03 | 4.81 | 1.81 | 4.83 | 1.91 |
| Positive affect (pre-study) | 3.81 | 1.89 | 3.81 | 1.90 | 3.25 | 1.72 | 3.27 | 1.60 | 4.15 | 1.96 | 4.63 | 1.80 | 4.38 | 1.75 |
| Moral self-appraisal | 5.21 | 2.02 | 4.80 | 2.20 | 5.68 | 1.99 | 5.74 | 1.95 | 6.25 | 1.70 | 6.34 | 1.60 | 5.89 | 1.84 |
| Autonomy: not obligated | 3.84 | 2.74 | 6.02 | 2.40 | 4.38 | 2.78 | 3.7 | 2.81 | 3.5 | 2.80 | 3.38 | 2.77 | 4.87 | 2.64 |
| Autonomy: want | 6.07 | 2.08 | 6.75 | 1.57 | 6.25 | 2.02 | 5.95 | 2.33 | 6.26 | 2.00 | 6.04 | 1.99 | 6.47 | 1.74 |
| Competence | 6.75 | 1.74 | 6.66 | 1.83 | 6.4 | 1.88 | 6.54 | 1.86 | 6.05 | 2.17 | 6.29 | 1.73 | 6.33 | 1.62 |
| Relatedness | 2.83 | 2.59 | 1.97 | 2.42 | 4.31 | 2.61 | 2.83 | 2.54 | 4.42 | 2.49 | 4.38 | 2.45 | 4.26 | 2.60 |

*Tables with Full Model Results*

**Table S4.3 Linear models of experimental effects on averaged content ratings from crowdsourced coders**. N = 1783. The reference category for experimental effects is the routine acts control condition. Coefficients are unstandardized. Heteroskedasticity-robust standard errors shown in parentheses. 95% confidence intervals shown in brackets. Estimates are adjusted for missing data using full information maximum likelihood.

|  | Response Content Ratings | | | | | | | | | | | | | | | | | | | | | | | | | | | | |
| --- | --- | --- | --- | --- | --- | --- | --- | --- | --- | --- | --- | --- | --- | --- | --- | --- | --- | --- | --- | --- | --- | --- | --- | --- | --- | --- | --- | --- | --- |
|  | *Care* | | | | |  | *Fairness* | | | | |  | *Loyalty* | | | | |  | *Authority* | | | | |  | *Sanctity* | | | | |
|  | **Est.** | **S.E.** | **p** | **95% CI** | |  | **Est.** | **S.E.** | **p** | **95% CI** | |  | **Est.** | **S.E.** | **p** | **95% CI** | |  | **Est.** | **S.E.** | **p** | **95% CI** | |  | **Est.** | **S.E.** | **p** | **95% CI** | |
| (Intercept) | 0.15 | (0.02) | <0.001 | [0.11, | 0.19] |  | 0.04 | (0.01) | <0.001 | [0.02, | 0.07] |  | 0.14 | (0.02) | <0.001 | [0.1, | 0.17] |  | 0.07 | (0.01) | <0.001 | [0.04, | 0.10] |  | 0.02 | (0.01) | 0.004 | [0.01, | 0.03] |
| *Experimental Conditions* | | |  |  |  |  |  |  |  |  |  |  |  |  |  |  |  |  |  |  |  |  |  |  |  |  |  |  |  |
| Self-indulgent | -0.05 | (0.02) | 0.059 | [-0.10, | 0.00] |  | -0.01 | (0.02) | 0.704 | [-0.04, | 0.02] |  | -0.03 | (0.02) | 0.184 | [-0.08, | 0.01] |  | -0.01 | (0.02) | 0.455 | [-0.05, | 0.02] |  | 0.01 | (0.01) | 0.385 | [-0.01, | 0.03] |
| Care | 0.51 | (0.04) | <0.001 | [0.44, | 0.58] |  | 0.01 | (0.02) | 0.769 | [-0.03, | 0.04] |  | 0.08 | (0.03) | 0.002 | [0.03, | 0.14] |  | -0.01 | (0.02) | 0.767 | [-0.05, | 0.03] |  | 0.00 | (0.01) | 0.898 | [-0.02, | 0.02] |
| Fairness | 0.11 | (0.03) | <0.001 | [0.05, | 0.17] |  | 0.40 | (0.03) | <0.001 | [0.34, | 0.46] |  | 0.06 | (0.03) | 0.029 | [0.01, | 0.11] |  | 0.00 | (0.02) | 0.916 | [-0.04, | 0.04] |  | 0.03 | (0.01) | 0.004 | [0.01, | 0.06] |
| Loyalty | 0.04 | (0.03) | 0.193 | [-0.02, | 0.10] |  | 0.05 | (0.02) | 0.013 | [0.01, | 0.09] |  | 0.41 | (0.04) | <0.001 | [0.35, | 0.48] |  | 0.00 | (0.02) | 0.990 | [-0.04, | 0.04] |  | 0.01 | (0.01) | 0.635 | [-0.02, | 0.03] |
| Authority | 0.00 | (0.03) | 0.903 | [-0.05, | 0.06] |  | 0.03 | (0.02) | 0.186 | [-0.01, | 0.06] |  | 0.11 | (0.03) | <0.001 | [0.06, | 0.17] |  | 0.30 | (0.03) | <0.001 | [0.24, | 0.36] |  | 0.01 | (0.01) | 0.341 | [-0.01, | 0.04] |
| Sanctity | 0.00 | (0.03) | 0.958 | [-0.06, | 0.05] |  | 0.01 | (0.02) | 0.439 | [-0.02, | 0.05] |  | 0.01 | (0.03) | 0.847 | [-0.05, | 0.06] |  | -0.01 | (0.02) | 0.591 | [-0.05, | 0.03] |  | 0.14 | (0.02) | <0.001 | [0.10, | 0.18] |
| *Controls (for randomization)* | | |  |  |  |  |  |  |  |  |  |  |  |  |  |  |  |  |  |  |  |  |  |  |  |  |  |  |  |
| MFQ:Care | -0.01 | (0.01) | 0.523 | [-0.03, | 0.01] |  | -0.01 | (0.01) | 0.353 | [-0.02, | 0.01] |  | 0.01 | (0.01) | 0.309 | [-0.01, | 0.03] |  | 0.00 | (0.01) | 0.696 | [-0.01, | 0.02] |  | 0.00 | (0.00) | 0.337 | [0.00, | 0.01] |
| MFQ:Fairness | 0.01 | (0.01) | 0.515 | [-0.01, | 0.03] |  | 0.00 | (0.01) | 0.923 | [-0.01, | 0.01] |  | -0.01 | (0.01) | 0.411 | [-0.03, | 0.01] |  | -0.01 | (0.01) | 0.455 | [-0.02, | 0.01] |  | -0.01 | (0.01) | 0.284 | [-0.02, | 0.00] |
| MFQ:Loyalty | 0.02 | (0.01) | 0.157 | [-0.01, | 0.04] |  | -0.01 | (0.01) | 0.464 | [-0.02, | 0.01] |  | 0.00 | (0.01) | 0.700 | [-0.03, | 0.02] |  | 0.01 | (0.01) | 0.562 | [-0.01, | 0.02] |  | 0.00 | (0.01) | 0.941 | [-0.01, | 0.01] |
| MFQ:Authority | -0.01 | (0.01) | 0.721 | [-0.03, | 0.02] |  | 0.01 | (0.01) | 0.365 | [-0.01, | 0.03] |  | 0.01 | (0.01) | 0.282 | [-0.01, | 0.04] |  | 0.02 | (0.01) | 0.151 | [-0.01, | 0.04] |  | 0.00 | (0.01) | 0.605 | [-0.01, | 0.02] |
| MFQ:Sanctity | -0.01 | (0.01) | 0.521 | [-0.03, | 0.02] |  | 0.01 | (0.01) | 0.540 | [-0.01, | 0.02] |  | 0.00 | (0.01) | 0.947 | [-0.02, | 0.02] |  | -0.01 | (0.01) | 0.138 | [-0.03, | 0.00] |  | 0.01 | (0.01) | 0.421 | [-0.01, | 0.02] |
| R^2^ | 0.21 |  |  |  |  |  | 0.23 |  |  |  |  |  | 0.16 |  |  |  |  |  | 0.15 |  |  |  |  |  | 0.09 |  |  |  |  |

**Table S4.4 Multiple equation linear model of experimental effects on positive affect (unmediated), moral self-appraisals, and ACR needs**. N = 1783. The reference category for experimental effects is the routine acts control condition. Moral self-appraisal, ACR needs, and positive affect variables are standardized. Heteroskedasticity-robust standard errors shown in parentheses. 95% confidence intervals shown in brackets. Estimates are adjusted for missing data using full information maximum likelihood.

|  | *Moral Self-Appraisals* | | | | |  | *Competence* | | | | |  | *Autonomy: not obligated* | | | | |  | *Autonomy: want* | | | | |  |
| --- | --- | --- | --- | --- | --- | --- | --- | --- | --- | --- | --- | --- | --- | --- | --- | --- | --- | --- | --- | --- | --- | --- | --- | --- |
|  | **Est.** | **S.E.** | **p** | **95% CI** | |  | **Est.** | **S.E.** | **p** | **95% CI** | |  | **Est.** | **S.E.** | **p** | **95% CI** | |  | **Est.** | **S.E.** | **p** | **95% CI** | |  |
| *Experimental Conditions* |  |  |  |  |  |  |  |  |  |  |  |  |  |  |  |  |  |  |  |  |  |  |  |  |
| Self-indulgent | -0.17 | (0.08) | 0.031 | [-0.32, | -0.02] |  | -0.04 | (0.08) | 0.595 | [-0.20, | 0.12] |  | 0.73 | (0.08) | <0.001 | [0.58, | 0.88] |  | 0.35 | (0.08) | <0.001 | [0.20, | 0.51] |  |
| Care | 0.55 | (0.08) | <0.001 | [0.40, | 0.71] |  | -0.19 | (0.09) | 0.026 | [-0.36, | -0.02] |  | 0.01 | (0.09) | 0.953 | [-0.17, | 0.18] |  | 0.12 | (0.09) | 0.214 | [-0.07, | 0.30] |  |
| Fairness | 0.58 | (0.08) | <0.001 | [0.42, | 0.74] |  | -0.12 | (0.09) | 0.164 | [-0.30, | 0.05] |  | -0.23 | (0.09) | 0.011 | [-0.40, | -0.05] |  | -0.04 | (0.10) | 0.696 | [-0.23, | 0.16] |  |
| Loyalty | 0.35 | (0.08) | <0.001 | [0.20, | 0.50] |  | -0.38 | (0.10) | <0.001 | [-0.57, | -0.19] |  | -0.01 | (0.09) | 0.923 | [-0.18, | 0.16] |  | 0.09 | (0.09) | 0.325 | [-0.09, | 0.27] |  |
| Authority | 0.28 | (0.07) | <0.001 | [0.14, | 0.43] |  | -0.28 | (0.08) | 0.001 | [-0.45, | -0.12] |  | -0.03 | (0.09) | 0.755 | [-0.2, | 0.14] |  | -0.06 | (0.09) | 0.548 | [-0.24, | 0.12] |  |
| Sanctity | 0.13 | (0.07) | 0.071 | [-0.01, | 0.27] |  | -0.23 | (0.08) | 0.004 | [-0.38, | -0.07] |  | 0.48 | (0.08) | <0.001 | [0.32, | 0.64] |  | 0.19 | (0.08) | 0.017 | [0.03, | 0.35] |  |
| *Mediators* |  |  |  |  |  |  |  |  |  |  |  |  |  |  |  |  |  |  |  |  |  |  |  |  |
| Moral self-appraisals |  |  |  |  |  |  |  |  |  |  |  |  |  |  |  |  |  |  |  |  |  |  |  |  |
| Competence |  |  |  |  |  |  |  |  |  |  |  |  |  |  |  |  |  |  |  |  |  |  |  |  |
| Autonomy: not obligated |  |  |  |  |  |  |  |  |  |  |  |  |  |  |  |  |  |  |  |  |  |  |  |  |
| Autonomy: want |  |  |  |  |  |  |  |  |  |  |  |  |  |  |  |  |  |  |  |  |  |  |  |  |
| Relatedness |  |  |  |  |  |  |  |  |  |  |  |  |  |  |  |  |  |  |  |  |  |  |  |  |
| MFQ:Care | 0.07 | (0.03) | 0.010 | [0.02, | 0.12] |  | 0.11 | (0.03) | <0.001 | [0.05, | 0.18] |  | -0.06 | (0.03) | 0.059 | [-0.11, | 0.00] |  | 0.15 | (0.03) | <0.001 | [0.09, | 0.22] |  |
| MFQ:Fairness | 0.10 | (0.03) | <0.001 | [0.04, | 0.15] |  | 0.08 | (0.03) | 0.010 | [0.02, | 0.14] |  | 0.00 | (0.03) | 0.942 | [-0.06, | 0.05] |  | 0.05 | (0.03) | 0.088 | [-0.01, | 0.11] |  |
| MFQ:Loyalty | -0.02 | (0.03) | 0.487 | [-0.09, | 0.04] |  | -0.08 | (0.04) | 0.015 | [-0.15, | -0.02] |  | -0.12 | (0.04) | 0.001 | [-0.19, | -0.05] |  | -0.12 | (0.04) | 0.001 | [-0.20, | -0.05] |  |
| MFQ:Authority | 0.14 | (0.04) | <0.001 | [0.07, | 0.22] |  | 0.10 | (0.04) | 0.017 | [0.02, | 0.17] |  | -0.03 | (0.04) | 0.483 | [-0.11, | 0.05] |  | 0.09 | (0.04) | 0.040 | [0.00, | 0.17] |  |
| MFQ:Sanctity | 0.08 | (0.03) | 0.014 | [0.02, | 0.15] |  | -0.10 | (0.04) | 0.005 | [-0.17, | -0.03] |  | -0.03 | (0.03) | 0.375 | [-0.10, | 0.04] |  | -0.03 | (0.04) | 0.397 | [-0.10, | 0.04] |  |
| Pre-study positive affect | 0.41 | (0.02) | <0.001 | [0.37, | 0.46] |  | 0.20 | (0.02) | <0.001 | [0.15, | 0.24] |  | -0.07 | (0.03) | 0.009 | [-0.12, | -0.02] |  | 0.18 | (0.03) | <0.001 | [0.13, | 0.23] |  |
| (Intercept) | -0.23 | (0.05) | <0.001 | [-0.33, | -0.13] |  | 0.17 | (0.06) | 0.002 | [0.06, | 0.28] |  | -0.15 | (0.06) | 0.008 | [-0.27, | -0.04] |  | -0.10 | (0.06) | 0.105 | [-0.22, | 0.02] |  |
| R^2^ | 0.30 |  |  |  |  |  | 0.09 |  |  |  |  |  | 0.13 |  |  |  |  |  | 0.09 |  |  |  |  |  |

(continued)

|  |  | *Relatedness* | | | | |  | *Positive Affect* | | | | |
| --- | --- | --- | --- | --- | --- | --- | --- | --- | --- | --- | --- | --- |
|  |  | **Est.** | **S.E.** | **p** | **95% CI** | |  | **Est.** | **S.E.** | **p** | **95% CI** | |
| *Experimental Conditions* |  |  |  |  |  |  |  |  |  |  |  |  |
| Self-indulgent |  | -0.28 | (0.07) | <0.001 | [-0.42, | -0.13] |  | 0.19 | (0.04) | <0.001 | [0.12, | 0.26] |
| Care |  | 0.77 | (0.08) | <0.001 | [0.61, | 0.94] |  | 0.13 | (0.04) | 0.002 | [0.05, | 0.21] |
| Fairness |  | 0.22 | (0.08) | 0.009 | [0.05, | 0.38] |  | 0.09 | (0.04) | 0.019 | [0.02, | 0.17] |
| Loyalty |  | 0.45 | (0.08) | <0.001 | [0.29, | 0.61] |  | 0.16 | (0.04) | <0.001 | [0.08, | 0.24] |
| Authority |  | 0.38 | (0.08) | <0.001 | [0.23, | 0.54] |  | 0.09 | (0.04) | 0.027 | [0.01, | 0.18] |
| Sanctity |  | 0.37 | (0.08) | <0.001 | [0.22, | 0.53] |  | 0.22 | (0.04) | <0.001 | [0.15, | 0.30] |
| *Mediators* |  |  |  |  |  |  |  |  |  |  |  |  |
| Moral self-appraisals |  |  |  |  |  |  |  |  |  |  |  |  |
| Competence |  |  |  |  |  |  |  |  |  |  |  |  |
| Autonomy: not obligated |  |  |  |  |  |  |  |  |  |  |  |  |
| Autonomy: want |  |  |  |  |  |  |  |  |  |  |  |  |
| Relatedness |  |  |  |  |  |  |  |  |  |  |  |  |
| MFQ:Care |  | 0.08 | (0.03) | 0.002 | [0.03, | 0.14] |  | 0.02 | (0.01) | 0.199 | [-0.01, | 0.05] |
| MFQ:Fairness |  | -0.02 | (0.03) | 0.533 | [-0.07, | 0.04] |  | 0.03 | (0.01) | 0.013 | [0.01, | 0.06] |
| MFQ:Loyalty |  | 0.14 | (0.04) | <0.001 | [0.07, | 0.21] |  | -0.02 | (0.02) | 0.423 | [-0.05, | 0.02] |
| MFQ:Authority |  | -0.03 | (0.04) | 0.475 | [-0.11, | 0.05] |  | 0.04 | (0.02) | 0.034 | [0.00, | 0.08] |
| MFQ:Sanctity |  | 0.06 | (0.03) | 0.088 | [-0.01, | 0.12] |  | -0.01 | (0.02) | 0.644 | [-0.04, | 0.03] |
| Pre-study positive affect |  | 0.24 | (0.02) | <0.001 | [0.19, | 0.28] |  | 0.86 | (0.01) | <0.001 | [0.84, | 0.89] |
| (Intercept) |  | -0.26 | (0.05) | <0.001 | [-0.37, | -0.16] |  | -0.13 | (0.02) | <0.001 | [-0.17, | -0.08] |
| R^2^ |  | 0.22 |  |  |  |  |  | 0.77 |  |  |  |  |

**Table S4.5 Multiple equation linear model of experimental effects on positive affect (mediated), moral self-appraisals, and ACR needs.** N = 1783. The reference category for experimental effects is the routine acts control condition. Moral self-appraisal, ACR needs, and positive affect variables are standardized. Heteroskedasticity-robust standard errors shown in parentheses. 95% confidence intervals shown in brackets. Estimates are adjusted for missing data using full information maximum likelihood.

|  | *Moral Self-Appraisals* | | | | |  | *Competence* | | | | |  | *Autonomy: not obligated* | | | | |  | *Autonomy: want* | | | | |  |
| --- | --- | --- | --- | --- | --- | --- | --- | --- | --- | --- | --- | --- | --- | --- | --- | --- | --- | --- | --- | --- | --- | --- | --- | --- |
|  | **Est.** | **S.E.** | **p** | **95% CI** | |  | **Est.** | **S.E.** | **p** | **95% CI** | |  | **Est.** | **S.E.** | **p** | **95% CI** | |  | **Est.** | **S.E.** | **p** | **95% CI** | |  |
| *Experimental Conditions* |  |  |  |  |  |  |  |  |  |  |  |  |  |  |  |  |  |  |  |  |  |  |  |  |
| Self-indulgent | -0.17 | (0.08) | 0.028 | [-0.32, | -0.02] |  | -0.04 | (0.08) | 0.615 | [-0.20, | 0.12] |  | 0.73 | (0.08) | <0.001 | [0.58, | 0.88] |  | 0.35 | (0.08) | <0.001 | [0.20, | 0.51] |  |
| Care | 0.56 | (0.08) | <0.001 | [0.40, | 0.71] |  | -0.19 | (0.09) | 0.028 | [-0.36, | -0.02] |  | 0.00 | (0.09) | 0.957 | [-0.17, | 0.18] |  | 0.12 | (0.09) | 0.211 | [-0.07, | 0.30] |  |
| Fairness | 0.59 | (0.08) | <0.001 | [0.43, | 0.75] |  | -0.12 | (0.09) | 0.170 | [-0.30, | 0.05] |  | -0.23 | (0.09) | 0.011 | [-0.40, | -0.05] |  | -0.04 | (0.10) | 0.698 | [-0.23, | 0.16] |  |
| Loyalty | 0.35 | (0.08) | <0.001 | [0.20, | 0.50] |  | -0.37 | (0.10) | <0.001 | [-0.56, | -0.18] |  | -0.01 | (0.09) | 0.913 | [-0.18, | 0.16] |  | 0.09 | (0.09) | 0.325 | [-0.09, | 0.27] |  |
| Authority | 0.28 | (0.07) | <0.001 | [0.14, | 0.43] |  | -0.28 | (0.08) | 0.001 | [-0.45, | -0.11] |  | -0.03 | (0.09) | 0.744 | [-0.20, | 0.14] |  | -0.05 | (0.09) | 0.557 | [-0.23, | 0.13] |  |
| Sanctity | 0.13 | (0.07) | 0.069 | [-0.01, | 0.27] |  | -0.22 | (0.08) | 0.005 | [-0.38, | -0.07] |  | 0.48 | (0.08) | <0.001 | [0.32, | 0.64] |  | 0.19 | (0.08) | 0.017 | [0.03, | 0.35] |  |
| *Mediators* |  |  |  |  |  |  |  |  |  |  |  |  |  |  |  |  |  |  |  |  |  |  |  |  |
| Moral self-appraisals |  |  |  |  |  |  |  |  |  |  |  |  |  |  |  |  |  |  |  |  |  |  |  |  |
| Competence |  |  |  |  |  |  |  |  |  |  |  |  |  |  |  |  |  |  |  |  |  |  |  |  |
| Autonomy: not obligated |  |  |  |  |  |  |  |  |  |  |  |  |  |  |  |  |  |  |  |  |  |  |  |  |
| Autonomy: want |  |  |  |  |  |  |  |  |  |  |  |  |  |  |  |  |  |  |  |  |  |  |  |  |
| Relatedness |  |  |  |  |  |  |  |  |  |  |  |  |  |  |  |  |  |  |  |  |  |  |  |  |
| MFQ:Care | 0.07 | (0.03) | 0.008 | [0.02, | 0.13] |  | 0.11 | (0.03) | <0.001 | [0.05, | 0.18] |  | -0.06 | (0.03) | 0.058 | [-0.12, | 0.00] |  | 0.16 | (0.03) | <0.001 | [0.09, | 0.22] |  |
| MFQ:Fairness | 0.09 | (0.03) | <0.001 | [0.04, | 0.15] |  | 0.08 | (0.03) | 0.010 | [0.02, | 0.14] |  | 0.00 | (0.03) | 0.942 | [-0.06, | 0.05] |  | 0.05 | (0.03) | 0.089 | [-0.01, | 0.11] |  |
| MFQ:Loyalty | -0.02 | (0.03) | 0.520 | [-0.09, | 0.04] |  | -0.08 | (0.04) | 0.016 | [-0.15, | -0.02] |  | -0.12 | (0.04) | 0.001 | [-0.19, | -0.05] |  | -0.12 | (0.04) | 0.001 | [-0.20, | -0.05] |  |
| MFQ:Authority | 0.14 | (0.04) | <0.001 | [0.07, | 0.22] |  | 0.10 | (0.04) | 0.016 | [0.02, | 0.18] |  | -0.03 | (0.04) | 0.474 | [-0.11, | 0.05] |  | 0.09 | (0.04) | 0.040 | [0.00, | 0.17] |  |
| MFQ:Sanctity | 0.08 | (0.03) | 0.016 | [0.02, | 0.15] |  | -0.10 | (0.04) | 0.005 | [-0.17, | -0.03] |  | -0.03 | (0.03) | 0.382 | [-0.10, | 0.04] |  | -0.03 | (0.04) | 0.385 | [-0.10, | 0.04] |  |
| Pre-study positive affect | 0.42 | (0.02) | <0.001 | [0.37, | 0.46] |  | 0.19 | (0.02) | <0.001 | [0.15, | 0.24] |  | -0.07 | (0.03) | 0.009 | [-0.12, | -0.02] |  | 0.18 | (0.03) | <0.001 | [0.13, | 0.23] |  |
| (Intercept) | -0.23 | (0.05) | <0.001 | [-0.33, | -0.13] |  | 0.17 | (0.06) | 0.002 | [0.06, | 0.28] |  | -0.15 | (0.06) | 0.009 | [-0.27, | -0.04] |  | -0.10 | (0.06) | 0.105 | [-0.22, | 0.02] |  |
| R^2^ | 0.31 |  |  |  |  |  | 0.08 |  |  |  |  |  | 0.13 |  |  |  |  |  | 0.09 |  |  |  |  |  |

(continued)

|  |  | *Relatedness* | | | | |  | *Positive Affect* | | | | |
| --- | --- | --- | --- | --- | --- | --- | --- | --- | --- | --- | --- | --- |
|  |  | **Est.** | **S.E.** | **p** | **95% CI** | |  | **Est.** | **S.E.** | **p** | **95% CI** | |
| *Experimental Conditions* |  |  |  |  |  |  |  |  |  |  |  |  |
| Self-indulgent |  | -0.28 | (0.07) | <0.001 | [-0.42, | -0.13] |  | 0.18 | (0.04) | <0.001 | [0.10, | 0.25] |
| Care |  | 0.77 | (0.08) | <0.001 | [0.61, | 0.94] |  | 0.03 | (0.04) | 0.472 | [-0.05, | 0.11] |
| Fairness |  | 0.22 | (0.08) | 0.009 | [0.06, | 0.38] |  | 0.03 | (0.04) | 0.418 | [-0.05, | 0.11] |
| Loyalty |  | 0.46 | (0.08) | <0.001 | [0.30, | 0.61] |  | 0.12 | (0.04) | 0.002 | [0.05, | 0.20] |
| Authority |  | 0.38 | (0.08) | <0.001 | [0.22, | 0.54] |  | 0.07 | (0.04) | 0.111 | [-0.02, | 0.15] |
| Sanctity |  | 0.37 | (0.08) | <0.001 | [0.22, | 0.53] |  | 0.17 | (0.04) | <0.001 | [0.10, | 0.25] |
| *Mediators* |  |  |  |  |  |  |  |  |  |  |  |  |
| Moral self-appraisals |  |  |  |  |  |  |  | 0.12 | (0.02) | <0.001 | [0.09, | 0.16] |
| Competence |  |  |  |  |  |  |  | 0.07 | (0.01) | <0.001 | [0.04, | 0.10] |
| Autonomy: not obligated |  |  |  |  |  |  |  | 0.05 | (0.01) | <0.001 | [0.03, | 0.07] |
| Autonomy: want |  |  |  |  |  |  |  | 0.03 | (0.01) | 0.014 | [0.01, | 0.06] |
| Relatedness |  |  |  |  |  |  |  | 0.04 | (0.01) | 0.001 | [0.02, | 0.07] |
| MFQ:Care |  | 0.08 | (0.03) | 0.002 | [0.03, | 0.14] |  | 0.00 | (0.01) | 0.718 | [-0.03, | 0.02] |
| MFQ:Fairness |  | -0.02 | (0.03) | 0.542 | [-0.07, | 0.04] |  | 0.02 | (0.01) | 0.224 | [-0.01, | 0.04] |
| MFQ:Loyalty |  | 0.14 | (0.04) | <0.001 | [0.07, | 0.21] |  | 0.00 | (0.02) | 0.881 | [-0.04, | 0.03] |
| MFQ:Authority |  | -0.03 | (0.04) | 0.489 | [-0.11, | 0.05] |  | 0.02 | (0.02) | 0.376 | [-0.02, | 0.05] |
| MFQ:Sanctity |  | 0.06 | (0.03) | 0.086 | [-0.01, | 0.13] |  | -0.01 | (0.02) | 0.506 | [-0.04, | 0.02] |
| Pre-study positive affect |  | 0.23 | (0.02) | <0.001 | [0.19, | 0.28] |  | 0.78 | (0.01) | <0.001 | [0.76, | 0.81] |
| (Intercept) |  | -0.26 | (0.05) | <0.001 | [-0.37, | -0.16] |  | -0.09 | (0.02) | <0.001 | [-0.13, | -0.04] |
| R^2^ |  | 0.22 |  |  |  |  |  | 0.80 |  |  |  |  |

**Table S4.6 Indirect effects from multiple equation linear model of experimental effects on positive affect (mediated), moral self-appraisals, and ACR needs**. See Table S4.5 for the full model. The reference category for indirect effects is the routine acts control condition. Bootstrapped 95% confidence intervals are shown (5000 replications).

| **Experimental Condition** |  |  |  | **95% Confidence Interval** | |
| --- | --- | --- | --- | --- | --- |
|  | **Mediator** | **Estimate** |  | **Lower** | **Upper** |
| Self-indulgent | Moral self-appraisal | -0.021 |  | -0.043 | -0.002 |
|  | Competence | -0.003 |  | -0.015 | 0.008 |
|  | Autonomy: not obligated | 0.037 |  | 0.018 | 0.057 |
|  | Autonomy: want | 0.012 |  | 0.002 | 0.024 |
|  | Relatedness | -0.012 |  | -0.023 | -0.004 |
| Care | Moral self-appraisal | 0.069 |  | 0.045 | 0.098 |
|  | Competence | -0.013 |  | -0.027 | -0.002 |
|  | Autonomy: not obligated | 0.000 |  | -0.009 | 0.010 |
|  | Autonomy: want | 0.004 |  | -0.002 | 0.013 |
|  | Relatedness | 0.035 |  | 0.014 | 0.057 |
| Fairness | Moral self-appraisal | 0.074 |  | 0.049 | 0.103 |
|  | Competence | -0.008 |  | -0.022 | 0.003 |
|  | Autonomy: not obligated | -0.011 |  | -0.023 | -0.002 |
|  | Autonomy: want | -0.001 |  | -0.010 | 0.005 |
|  | Relatedness | 0.010 |  | 0.002 | 0.021 |
| Loyalty | Moral self-appraisal | 0.043 |  | 0.023 | 0.068 |
|  | Competence | -0.026 |  | -0.045 | -0.011 |
|  | Autonomy: not obligated | 0.000 |  | -0.010 | 0.009 |
|  | Autonomy: want | 0.003 |  | -0.003 | 0.011 |
|  | Relatedness | 0.020 |  | 0.008 | 0.036 |
| Authority | Moral self-appraisal | 0.035 |  | 0.016 | 0.057 |
|  | Competence | -0.019 |  | -0.036 | -0.007 |
|  | Autonomy: not obligated | -0.001 |  | -0.011 | 0.008 |
|  | Autonomy: want | -0.002 |  | -0.010 | 0.004 |
|  | Relatedness | 0.017 |  | 0.006 | 0.030 |
| Sanctity | Moral self-appraisal | 0.016 |  | -0.001 | 0.036 |
|  | Competence | -0.016 |  | -0.031 | -0.004 |
|  | Autonomy: not obligated | 0.024 |  | 0.011 | 0.040 |
|  | Autonomy: want | 0.006 |  | 0.000 | 0.015 |
|  | Relatedness | 0.017 |  | 0.006 | 0.030 |
