# Supplementary material for: Beyond prosociality: Recalling many types of moral behavior produces positive emotion
Source: PLoS One. 2022 Nov 11;17(11):e0277488. doi: 10.1371/journal.pone.0277488 (PMC9651559; doi:10.1371/journal.pone.0277488)
Supplement: S5 Appendix — (DOCX) [file pone.0277488.s005.docx]

**Supporting information 5: Effect Sizes and Morality as a Basic Psychological Need**

The main text presents effect sizes for our five morality conditions using *y*-standardized coefficients, which in this case are very similar to Cohen’s *d* scores. An alternative is to examine what proportion of research participants exhibited higher levels of positive affect following the recall task. To do this, we subtracted (unstandardized) pre-study positive affect from (unstandardized) post-experimental positive affect. We rounded the resulting change scores to the nearest whole number to guard against small fluctuations around 0 that might not reflect true change. We then calculated the proportion of respondents who increased, decreased, or exhibited no change in positive affect in each experimental condition. The results are shown in table S5.1.

Table S5.1 shows that roughly 10% to 15% of respondents decreased in positive affect across all conditions, and about half of respondents in each condition showed no change, though the routine acts condition showed a slightly higher rate of 61%. The main differences between conditions appear among those who increased in positive affect. In the routine acts control condition, 22% of respondents increased in positive affect, while in four out of five moral recall conditions that figure is 34% or 35%. Results for the sanctity and self-indulgent purchase conditions are even higher, with 40% of respondents increasing in positive affect. This means that those recalling moral acts were anywhere from 55% to 82% more likely than controls to increase in positive affect.

**Table S5.1 Changes in positive affect**. Numbers are proportions of respondents.

|  | **Decrease** | **No change** | **Increase** |
| --- | --- | --- | --- |
| Routine acts (control) | 0.17 | 0.61 | 0.22 |
| Self-indulgent | 0.11 | 0.47 | 0.42 |
| Care | 0.13 | 0.52 | 0.35 |
| Fairness | 0.15 | 0.51 | 0.34 |
| Loyalty | 0.10 | 0.56 | 0.34 |
| Authority | 0.18 | 0.47 | 0.35 |
| Sanctity | 0.08 | 0.52 | 0.40 |

While Table S5.1 makes it clear that those recalling moral or self-indulgent behaviors were more likely to increase in positive affect, it is nonetheless striking that across conditions only a minority of respondents showed any gains. At first glance, this seems to contradict the idea that morality is a basic psychological need because one hallmark of basic psychological needs is that satisfying them universally produces emotional well-being [1]. However, there are at least two possible interpretations of these results which have different implications for the hypothesis that morality is a basic psychological need.

The first is that moral self-appraisals have a positive effect for all respondents, but that in any given situation these effects might be counteracted by other predictors of positive affect or idiosyncratic features of the person or situation. Practically, this means that in any single experiment we should not expect that moral self-appraisals increase positive affect for all respondents. However, the moral self-appraisal effects should appear for everyone if we view them across situations and include appropriate controls for countervailing forces in our models. This pattern of results would suggest a universal effect of moral self-appraisals and would therefore be consistent with the morality-as-a-need hypothesis.

The second possibility is that moral self-appraisals only increase positive emotions for some people. In a single experiment, this would lead to the same pattern of results as before—that is, that moral self-appraisals only predict positive affect for some respondents. However, this pattern would persist even if we viewed respondents across situations and included appropriate controls for countervailing forces. Because basic psychological needs are universal features of human psychology, this pattern of results would not be consistent with the morality-as-a-need hypothesis.

The fact that there are two possible interpretations for the presence of positive, negative, and no change effects in our study means that our results tell us little about whether moral self-appraisal effects are universal. Our discussion does, however, suggest two ways to rigorously test this claim in future research.

First, researchers can use interaction terms in their statistical models to test whether moral self-appraisal effects vary across different types of people (e.g., across race, class, gender, or nationality). Two patterns of results would lend credence to the claim that moral self-appraisal effects do not vary across individuals: 1) a lack of sizable and statistically significant interaction terms, or 2) a positive estimated effect of moral self-appraisals for all respondents even in the presence of statistically significant interaction terms. One advantage of this approach is that it can be conducted on data collected at a single time point. However, this sort of analysis would always be vulnerable to the possibility that moral self-appraisal effects would vary by interpersonal differences that were not measured.

Second, researchers can observe the same individuals across contexts and over time. This should capture a wide variety of situations and increase the chance that any moral self-appraisal effects have an opportunity to manifest. Multilevel models with random effects for moral self-appraisals can then be used to provide individual estimates of the effect of moral self-appraisals on positive emotions. If all of these estimates are positive, then we would have strong evidence that moral self-appraisals enhance emotional well-being for everyone in the sample and—assuming the sample is generalizable to a larger population—people in the population generally.

This second approach strikes us as particularly promising, so we present the basic model in equation form in hopes of stimulating future research:

$$y_{it}=\gamma_{00}+\gamma_{10}x_{it}+U_{0i}+U_{1i}x_{it}+e_{it}$$

Here $y_{it}$ is positive affect which can vary across both individuals (*i*) and time (*t*). $\gamma_{00}$ is the average level of positive affect aggregated across individuals and time, and $U_{0i}$ are individual variations from that average. $x_{it}$ are moral self-appraisals that can vary over both individuals and time. Their coefficient, $\gamma_{10}$, is the average effect of moral self-appraisals in the sample. Random slopes for moral self-appraisals are given by $U_{1i}x_{it}$, which allows the effect of moral self-appraisals to vary across individuals. $e_{it}$ captures idiosyncratic influences on positive affect that can vary across both individuals and time.

This model has been left simple for expository simplicity. In practice, analysts would likely want to add controls for potential confounds of the moral self-appraisal effect. Additionally, the model could be estimated using fixed effects estimation to provide additional protection from omitted variables [2].

**S5 References**

1. Ryan RM, Deci EL. Self-Determination Theory: Basic Psychological Needs in Motivation, Development, and Wellness. New York: Guilford Press; 2018.

2. Allison PD. Fixed Effects Regression Models. Los Angeles, C.A.: Sage; 2009.
