# Supplementary material for: Beyond prosociality: Recalling many types of moral behavior produces positive emotion
Source: PLoS One. 2022 Nov 11;17(11):e0277488. doi: 10.1371/journal.pone.0277488 (PMC9651559; doi:10.1371/journal.pone.0277488)
Supplement: S6 Appendix — (DOCX) [file pone.0277488.s006.docx]

**Supporting information 6: Comparison of Estimates for Moral Recall and Self-Indulgent Purchase Conditions**

In the main text we compared estimates for the moral recall conditions to those from the routine acts condition. Here, we compare them to the self-indulgent purchase condition.

Fig. 4 (in the main text) shows that recalling any type of moral act (except sanctity) had a smaller estimated effect than recalling purchasing something that the respondent really wanted. However, these differences are only significant at the 0.05 level for fairness (*b* = -0.09 [-0.18, -0.01], *p* = 0.035) and authority (*b* = -0.09 [-0.19, -0.00], *p* = 0.048), and not for care (*b* = -0.06 [-0.15, 0.02], *p* = 0.157) or loyalty (*b* = -0.02 [-0.11, 0.06], *p* = 0.589).

These results could suggest that some of the observed differences do not actually exist, and that moral behaviors have similar effects on positive emotions as self-indulgent purchases. However, given the consistently smaller estimated effects among moral recall conditions, we think it more likely that recalling moral acts genuinely generates less positive affect under conditions like those in our study. We cannot, however, conclude that moral acts will *always* be less effective that self-indulgent acts. Both moral acts and self-indulgent purchases can vary in degree, with varying degrees plausibly leading to different levels of emotional benefit. Our experimental prompts did not match the degree between moral acts and self-indulgent purchases. For instance, asking someone to report the last time they intentionally helped or cared for someone else might evoke acts that are large or small, and that consequently might evoke emotions of varying intensity. However, recalling a purchase of something that a person really wanted necessarily evokes a high level of positive emotion. How moral and self-indulgent acts of equal degree compare in their ability to generate positive emotions remains an open question.
