# Supplementary material for: Beyond prosociality: Recalling many types of moral behavior produces positive emotion
Source: PLoS One. 2022 Nov 11;17(11):e0277488. doi: 10.1371/journal.pone.0277488 (PMC9651559; doi:10.1371/journal.pone.0277488)
Supplement: S7 Appendix — (DOCX) [file pone.0277488.s007.docx]

**Supporting information 7: Alternate Coding of Moral Self-Appraisals**

The analyses presented in the main text use the maximum amount of information available to measure ACR needs and moral self-appraisals. However, this leads to a possible imbalance in the accuracy with which each construct was measured. Because poor measurement can reduce effect sizes, this imbalance makes it problematic to directly compare the indirect effects through each of these constructs (see Figure 5).

In this supporting information, we address this issue by re-estimating the mediation model from the main text using a measure of moral self-appraisals that has the same number of items as our measures of autonomy and relatedness—two. We test two different versions of a two-item measure. Recall first that moral self-appraisals were measured by asking respondents how well different traits described them, based on how they acted during the recalled event. Our original measure included ratings of “moral”, “principled”, “upright”, and “good”. To develop a two-item measure, we first opted to include the “moral” item for reasons of face validity, and to not include “upright” because it strikes us as being a less common term, and hence less likely to be used by respondents to describe themselves. This left us with two combinations: “moral” and “principled,” and “moral” and “good”. Both of these 2-item scales have a reliability of α = 0.80.

Indirect effects from the original mediation model (Figure 5 and Table S4.6) and the models using the two 2-item scales are shown in Table S7.1. Using either of the two-item versions leads to some fluctuations in the indirect effects, but for those in the care, fairness, loyalty, and authority conditions the indirect effects through moral self-appraisals remain the largest positive effects, and in most cases the largest effects overall. The strength of the indirect effect through moral self-appraisals relative to the indirect effects through ACR needs thus persists even when the item-count measurement advantage of moral self-appraisals is removed.

**Table S7.1 Comparison of indirect effects from models using the original coding of moral-self appraisals, and two two-item versions**.

| **Experimental Condition** | **Mediator** | **Original Est.** |  | **Moral/**  **Principled** |  | **Moral/ Good** |
| --- | --- | --- | --- | --- | --- | --- |
| Self-indulgent | Moral self-appraisal | -0.021 |  | -0.043 |  | -0.002 |
|  | Competence | -0.003 |  | -0.015 |  | 0.008 |
|  | Autonomy: not obligated | 0.037 |  | 0.018 |  | 0.057 |
|  | Autonomy: want | 0.012 |  | 0.002 |  | 0.024 |
|  | Relatedness | -0.012 |  | -0.023 |  | -0.004 |
| Care | Moral self-appraisal | 0.069 |  | 0.045 |  | 0.098 |
|  | Competence | -0.013 |  | -0.027 |  | -0.002 |
|  | Autonomy: not obligated | 0.000 |  | -0.009 |  | 0.010 |
|  | Autonomy: want | 0.004 |  | -0.002 |  | 0.013 |
|  | Relatedness | 0.035 |  | 0.014 |  | 0.057 |
| Fairness | Moral self-appraisal | 0.074 |  | 0.049 |  | 0.103 |
|  | Competence | -0.008 |  | -0.022 |  | 0.003 |
|  | Autonomy: not obligated | -0.011 |  | -0.023 |  | -0.002 |
|  | Autonomy: want | -0.001 |  | -0.010 |  | 0.005 |
|  | Relatedness | 0.010 |  | 0.002 |  | 0.021 |
| Loyalty | Moral self-appraisal | 0.043 |  | 0.023 |  | 0.068 |
|  | Competence | -0.026 |  | -0.045 |  | -0.011 |
|  | Autonomy: not obligated | 0.000 |  | -0.010 |  | 0.009 |
|  | Autonomy: want | 0.003 |  | -0.003 |  | 0.011 |
|  | Relatedness | 0.020 |  | 0.008 |  | 0.036 |
| Authority | Moral self-appraisal | 0.035 |  | 0.016 |  | 0.057 |
|  | Competence | -0.019 |  | -0.036 |  | -0.007 |
|  | Autonomy: not obligated | -0.001 |  | -0.011 |  | 0.008 |
|  | Autonomy: want | -0.002 |  | -0.010 |  | 0.004 |
|  | Relatedness | 0.017 |  | 0.006 |  | 0.030 |
| Sanctity | Moral self-appraisal | 0.016 |  | -0.001 |  | 0.036 |
|  | Competence | -0.016 |  | -0.031 |  | -0.004 |
|  | Autonomy: not obligated | 0.024 |  | 0.011 |  | 0.040 |
|  | Autonomy: want | 0.006 |  | 0.000 |  | 0.015 |
|  | Relatedness | 0.017 |  | 0.006 |  | 0.030 |
