# Supplementary material for: Beyond prosociality: Recalling many types of moral behavior produces positive emotion
Source: PLoS One. 2022 Nov 11;17(11):e0277488. doi: 10.1371/journal.pone.0277488 (PMC9651559; doi:10.1371/journal.pone.0277488)
Supplement: S8 Appendix — (DOCX) [file pone.0277488.s008.docx]

**Supporting information 8: Details on randomization procedure**

As noted in the main manuscript, we assigned respondents to experimental conditions based on their scores on the 30 item Moral Foundations Questionnaire (MFQ30). Full details are given below.

1. We ran a power analysis to determine the necessary sample size. Based on this analysis, we determined that we needed 234 per condition, and aimed to collect roughly 285 per condition to safeguard against unusable responses. This gave us an overall target sample size of about N = 2000.
2. Based on prior work (others’ and our own), we realized that most people would strongly endorse care and fairness moral foundations, but only a subset of respondents would strongly endorse loyalty, authority, and sanctity. To ensure that respondents received experimental prompts that were meaningful to them, we decided to randomize based on MFQ30 scores so that those high in loyalty, authority, and sanctity would be more likely to end up in the loyalty, authority, and sanctity experimental conditions. We had two randomization “tracks”. Those who scored above a certain cutoff value on the MFQ30 subscales for loyalty, authority, or sanctity would be randomly assigned to one of those conditions or to one of the comparison conditions (grocery store or self-indulgent purchase). Those who scored below the cutoff would be randomly assigned to the care, fairness, or one of the comparison conditions.
3. This randomization scheme had two sets of parameters that needed to be determined. First was the cutoff score for sorting people in the loyalty/authority/sanctity or the care/fairness track. The second parameters were the probabilities with which people in each track would be assigned to one of the experimental conditions. The goal in adjusting these two sets of parameters was to achieve about 285 respondents per experimental condition.
4. We used simulations to determine the two sets of parameters. First, we randomly generated samples of respondents (of different sizes) based on the means and covariances of MFQ30 subscales taken from an earlier sample we had collected using the same population (Amazon’s Mechanical Turk workers). The vector of means and matrix of covariances (correlations) used to generate samples is shown below. The order of subscales in both cases is: care, fairness, loyalty, authority, sanctity.

$$\mu=\left[ 3.78, 3.72, 2.50, 2.70, 2.25 \right]$$

$$\Sigma=\left[ \begin{matrix} 1 & & & & \\ 0.59 & 1 & & & \\ 0.20 & 0.05 & 1 & & \\ 0.20 & 0.06 & 0.76 & 1 & \\ 0.20 & 0.00 & 0.60 & 0.68 & 1 \end{matrix} \right]$$

5. In each simulated sample, we tested candidate cutoff values and assignment probabilities until we found a combination that returned around 285 respondents per condition.
6. Based on these simulations we chose 3.8 for the cutoff value (2.8 in the original simulation, but it used MFQ30 scores ranging from 0 to 5 instead of 1 to 6, which is what we used in the final data collection). We chose assignment probabilities in the loyalty/authority/purity track of: *p*_loyal_ = 0.25, *p*_auth_ = 0.25, *p*_sanctity_ = 0.25, *p*_grocery_ = 0.125, *p*_indulge_ = 0.125. We chose assignment probabilities in the care/fairness track of: *p*_care_ = 0.35, *p*_fairness_ = 0.35, *p*_grocery_ = 0.15, *p*_indulge_ = 0.15. To simplify the coding when programming the experiment into software, we changed the care/fairness probabilities to: *p*_care_ = 0.33, *p*_fairness_ = 0.33, *p*_grocery_ = 0.167, *p*_indulge_ = 0.167.

The R code we used to perform the power analysis and simulations is available at <https://osf.io/udphs/>.
